# Heterostructured photocatalysts in the removal of methylene blue dye: Mechanisms, recent advances, and design principles

**DOI:** 10.1016/j.isci.2026.117296

**Published:** 2026-08-25

**Authors:** Damilola Caleb Akintayo, Benjamin O. Orimolade, Adedamola T. Ojedokun, Mercy Ola, Tunde Lewis Yusuf

**Affiliations:** 1 Department of Chemistry, Faculty of Natural and Agricultural Sciences, University of Pretoria, Private Bag X20, Hatfield, Pretoria 0028, South Africa

**Keywords:** S-scheme heterojunction, Z-scheme, charge transfer mechanism, reactive oxygen species, methylene blue

## Abstract

Methylene blue (MB) is a persistent dye pollutant widely used to evaluate photocatalytic wastewater treatment because of its environmental persistence and well-defined degradation behavior. This review examines the design principles, charge-transfer mechanisms, and performance of more than 50 binary and ternary heterostructured photocatalysts, including Type II, Z-scheme, S-scheme, p-n, and Schottky architectures based on TiO_2_, ZnO, bismuth-based semiconductors, g-C_3_N_4_, and MXene-containing composites. Comparative analysis shows that S-scheme heterojunctions best preserve the redox potentials required for reactive oxygen species generation, while pore architecture strongly influences adsorption and degradation kinetics. The review also demonstrates that decolorization at 664 nm frequently overestimates treatment efficiency because organic intermediates can remain after color removal, thereby highlighting the importance of total organic carbon measurements. These findings provide practical guidance for photocatalyst design, mechanistic verification, and standardized performance assessment to support the development of more effective solar-driven wastewater treatment technologies.

## Graphical abstract

**Figure:**
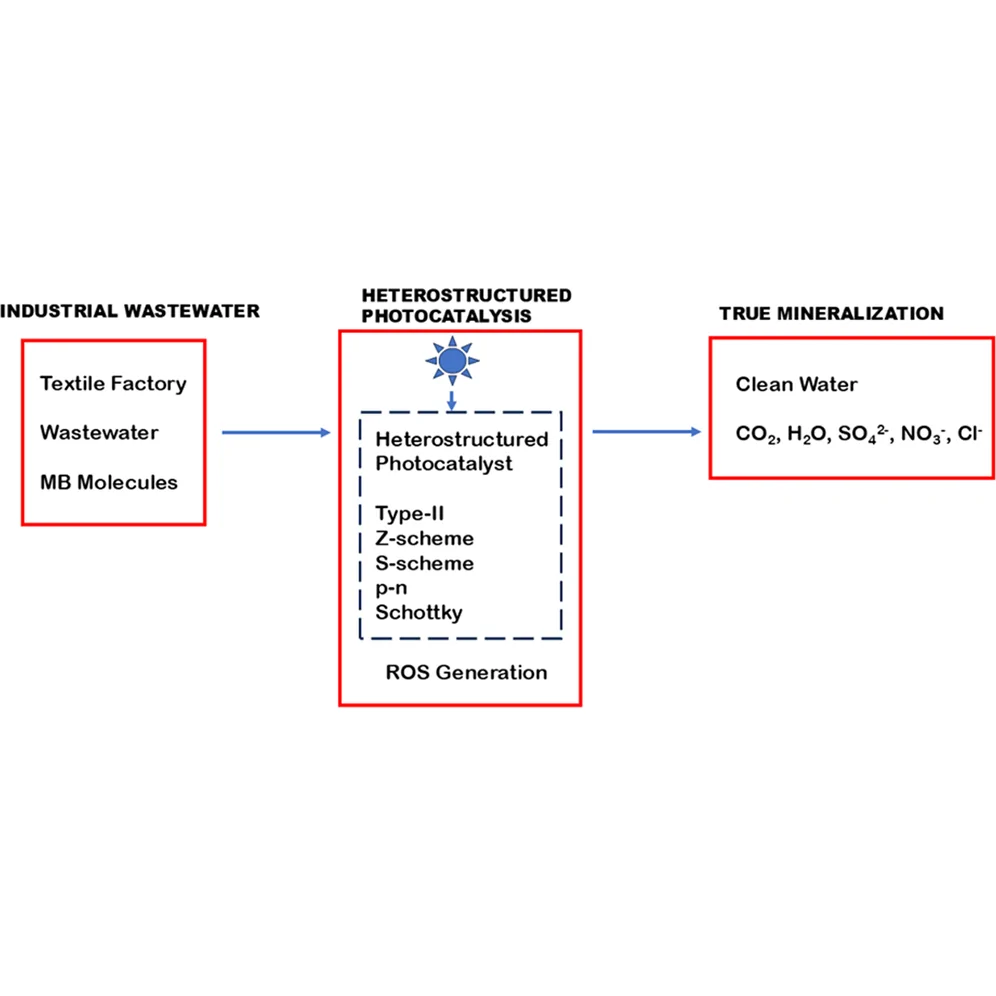

## Teaser

Catalysis; Semiconductor manufacturing; Materials science

## Introduction

The discharge of synthetic dyes from textile, printing, leather, pharmaceutical, and cosmetic industries remains a major source of water pollution worldwide. Among these dyes, methylene blue (MB) is one of the most extensively studied due to its widespread use and environmental persistence. MB is a cationic phenothiazine dye that is used in textile dyeing, biological staining, and medical treatments such as the management of methemoglobinemia.^1^^,^^2^^,^^3^ Its resistance to biological and photolytic degradation, strong absorption at 664 nm, and toxicity to aquatic organisms and humans at concentrations as low as 0.05 mg/L have made it a priority environmental contaminant and a standard model pollutant in photocatalytic research.^4^^,^^5^ Textile effluents may contain MB concentrations of up to 50 mg/L, which can inhibit photosynthesis, disrupt aquatic reproduction, and degrade the ecological quality of receiving water bodies.^6^

Traditional treatment technologies, including coagulation-flocculation, activated carbon adsorption, and membrane filtration,^7^^,^^8^^,^^9^^,^^10^ often fail to achieve complete mineralization of MB. In many cases, these methods generate secondary waste streams, especially sludge, thereby transferring contaminants from water to solid residues rather than eliminating them.^11^ On the other hand, heterogeneous photocatalysis offers the possibility of complete degradation by generating reactive oxygen species (ROS) from light-excited semiconductors, converting MB into CO_2_, H_2_O, and inorganic ions under mild operating conditions. However, conventional photocatalysts such as TiO_2_ and ZnO are limited by their wide bandgaps (∼3.2 eV), which restrict light absorption primarily to the ultraviolet region, representing only about 4% of solar energy, and by rapid electron-hole recombination that reduces photocatalytic efficiency.^12^ Although visible-light-responsive materials such as BiVO_4_, WO_3_, Bi_2_WO_6_, and g-C_3_N_4_ overcome the spectral limitation, they still suffer from significant charge-carrier recombination.^13^^,^^14^ To address these challenges, heterostructured photocatalysts have been developed to enhance visible-light utilization while promoting efficient charge separation through interfacial charge transfer processes.^15^^,^^16^

As a result, research on heterostructured photocatalysts for MB degradation has expanded rapidly, accompanied by numerous review articles. Nevertheless, important gaps remain. Broad reviews of heterojunction photocatalysts^17^^,^^18^^,^^19^^,^^20^ often draw generalized conclusions across diverse pollutant classes despite substantial differences in degradation behavior and ROS requirements. Other MB-related reviews have focused mainly on adsorption materials, including magsorbents, bio-waste biosorbents, carbon-based adsorbents, hydrogels, and gold nanoparticles.^21^^,^^22^^,^^23^^,^^24^^,^^25^ Consequently, they do not address key photocatalytic aspects such as charge-transfer mechanisms, ROS generation, and mineralization pathways. Recent reviews of bismuth-based ternary heterojunctions and black phosphorus heterostructures^26^^,^^27^ provide valuable insights into related areas but do not offer a comprehensive analysis of heterostructures specifically designed for MB degradation. Similarly, the review by Lai and Lee focuses exclusively on mediator Z-scheme systems and does not compare alternative heterojunction architectures or critically evaluate the evidence used to distinguish Z-scheme from Type II mechanisms.^17^^,^^18^^,^^19^^,^^20^

In this review, we examine the mechanistic principles, structural design, and photocatalytic performance of heterostructured materials developed for MB removal. We evaluate more than 50 binary and ternary systems spanning Type II, Z-scheme, S-scheme, p–n, and Schottky junction architectures, with material coverage extending across TiO_2_-, ZnO-, bismuth-based, g-C_3_N_4_-, and MXene-integrated platforms. The review begins with the physicochemical properties and environmental significance of MB, followed by a critical assessment of heterojunction charge-transfer mechanisms and the characterization standards necessary to distinguish between them reliably. We then examine the performance of each material family, analyze the influence of pore architecture on adsorption-degradation kinetics, and interrogate the decolorization-mineralization gap that causes UV–Vis-based removal efficiency to overestimate true photocatalytic performance. The review concludes with a critical appraisal of scalability, precursor cost, and the operational barriers that currently prevent these materials from advancing beyond the laboratory.

## Methylene blue: properties, environmental occurrence, and relevance as a benchmark pollutant

### Physicochemical properties and industrial applications

**Figure 1:**
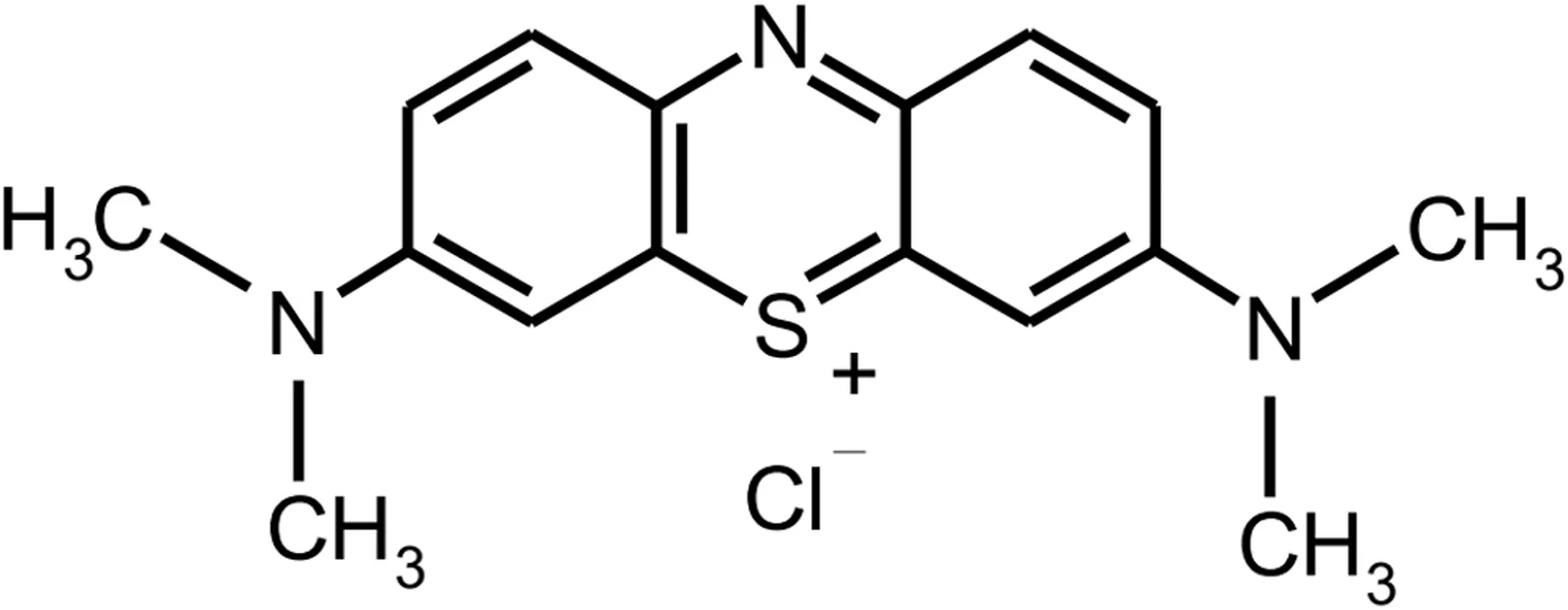
Chemical structure of methylene blue

MB, with the chemical formula C_16_H_18_ClN_3_S (Figure 1 shows its chemical structure), is a synthetic dye belonging to the phenothiazine class. It is a heterocyclic aromatic compound with a planar structure and has a molecular weight of 319.85 g/mol.^28^ It has a long-standing presence in industrial and medical applications due to its vibrant blue coloration and stable physicochemical properties. MB is used extensively in dyeing processes for textiles such as cotton and wool, in leather treatment, and as a staining agent in laboratory settings.^29^ It is also used medically as a diagnostic dye, in pharmaceutical development, and occasionally in therapeutic contexts, including as an antidote for methemoglobinemia.^1^^,^^2^^,^^3^ MB is characterized by its intense absorption in the visible light region, with a maximum absorbance at approximately 664 nm. This property makes it a commonly chosen model compound in photocatalytic degradation studies.^30^^,^^31^ Its molecular structure includes aromatic rings and nitrogen-containing functional groups that confer high chemical and photostability, as well as resistance to microbial degradation. MB is highly soluble in water and maintains its structural integrity under ambient environmental conditions, making it a persistent contaminant.^5^^,^^32^

### Environmental occurrence and ecotoxicological significance

The widespread industrial use of MB has resulted in its frequent presence in wastewater, especially in effluents from textile manufacturing and dyeing operations. In regions without stringent environmental regulations or adequate wastewater treatment infrastructure, these effluents are often discharged directly into water bodies. In addition to industrial sources, laboratories and healthcare facilities contribute to localized contamination through improper disposal practices. MB is commonly detected in wastewater at concentrations ranging from a few micrograms per liter (μg/L) to tens of milligrams per liter (mg/L). For instance, untreated textile effluents may contain MB levels of up to 50 mg/L, while environmental samples collected downstream of discharge points may show persistent traces in the range of 0.05–1.0 mg/L.^5^^,^^6^ These concentrations, even at their lower end, are sufficient to impart visible discoloration to natural water bodies, significantly affecting their aesthetic appeal and recreational value.

Beyond its visual impact, MB has been shown to interfere with ecological processes, especially in aquatic environments. Its presence in surface waters reduces light penetration, thereby inhibiting photosynthetic activity in aquatic plants and algae. This disruption cuts through the food web, affecting primary productivity and the overall health of aquatic ecosystems. Toxicological studies further indicate that MB can induce oxidative stress in aquatic organisms, interfere with metabolic and enzymatic pathways, and impair reproduction in fish and invertebrates. In human populations, exposure to MB-contaminated water may lead to adverse effects such as vomiting, dizziness, central nervous system disturbances, and in more severe cases, methemoglobinemia.^4^^,^^7^^,^^33^^,^^34^

Due to its chemical stability, resistance to biodegradation, and well-documented ecological and health impacts, MB is classified as a priority pollutant. Its persistence in the environment highlights the need for efficient removal technologies that go beyond conventional treatment. MB’s consistent use as a benchmark dye in photocatalytic studies underscores both its environmental significance and its utility in evaluating advanced treatment systems. In particular, photocatalysts that operate effectively under visible light are of interest, given their potential for solar-driven applications. Among these, heterostructured photocatalysts have shown considerable promise in addressing the limitations of single-component materials and enhancing degradation efficiency.

### MB as a benchmark pollutant

Despite its widespread use as a model pollutant in photocatalytic research, MB presents several limitations that the field has been slow to acknowledge. A fundamental concern is the conflation of decolorization with degradation, and degradation with mineralization. Most studies in the heterostructured photocatalysis literature report removal efficiency based exclusively on the attenuation of absorbance at 664 nm. While this metric is convenient and widely adopted, it measures decolorization, not degradation, and certainly not mineralization. Decolorization occurs when the chromophoric functional groups of the MB molecule are disrupted, abolishing visible color, but substantial quantities of colorless organic fragments and potentially toxic intermediates can persist in solution well after the 664 nm signal has vanished. This distinction has profound implications for environmental safety and for the honest evaluation of photocatalytic performance.

MB is a phenothiazine-based cationic dye with a complex aromatic core that includes a central heterocyclic ring system flanked by two dimethylamino-substituted benzene rings bridged by a sulfur atom. Its degradation by ROS, predominantly ·OH, ·O_2_^−^, and h^+^, does not proceed in a single step but through a cascade of sequential oxidative transformations that progressively reduce the molecular complexity of the parent compound. The degradation proceeds through a cascade of sequential oxidative transformations (initiated by demethylation and sulfoxide formation, followed by ring rupture and fragmentation to low-molecular-weight aliphatic intermediates), thereby yielding CO_2_, H_2_O, and inorganic ions (NH_4_^+^/NO_3_^−^, SO_4_^2−^, Cl^−^) upon complete mineralization. The mechanistic detail of this cascade, including the identity and toxicological significance of each intermediate class, is presented in full in Section MB degradation pathways, intermediate products, and mineralization extent.

The practical distinction between decolorization, degradation, and complete mineralization is captured quantitatively by TOC and COD measurements. A consistent pattern emerges in systems where these metrics have been reported alongside UV-Vis data. TOC removal lags behind decolorization. For example, in a study using a CeO_2_ nanoparticle/graphene oxide/polyacrylamide composite, 90% MB decolorization under UV-A light was accompanied by TOC and COD removal values that trailed the decolorization endpoint, confirming that a meaningful fraction of the original organic carbon persisted as colorless intermediates after the 664 nm signal had disappeared.^35^ Similarly, a Mg-Al LDH@g-C_3_N_4_@Ag_3_PO_4_ ternary heterostructure achieved 99% MB removal efficiency but only 88% TOC removal, thereby demonstrating that even highly efficient decolorization systems leave behind non-trivial quantities of persistent organic intermediates.^36^ These findings show that color removal consistently overestimates the true extent of mineralization.

These considerations carry direct and actionable implications for the evaluation of heterostructured photocatalysts throughout this review. The efficiency data compiled in Table 1 (based almost entirely on absorbance at 664 nm) reflect decolorization rather than mineralization, and the true environmental efficacy of the reviewed systems likely varies more widely than those numbers suggest. The superior radical generation capacity of Z-scheme and S-scheme heterostructures, which preserve high-energy electrons and holes, should in principle translate into deeper mineralization compared to Type II systems in which transferred carriers have reduced redox potentials. However, this hypothesis remains untested because TOC analyses are so rarely performed.

**Table 1:** Summary of recent studies on the photocatalytic removal of MB using heterostructures

Photocatalyst composition	Heterojunction type	Photocatalysis condition	MB removal (%)	Apparent rate constant (k, min^−1^)	Key features	Reference
Ag_3_PO_4_/N–TiO_2_ nanotubes	Type II	5 mg/L MB, 75 mL, 0.5 g/L catalyst, 500 W Xe lamp	83% in 3 h	0.0098	N-doping and Ag_3_PO_4_ extend visible activity and promote charge separation	Luan et al.^37^
TiO_2_–CdS/BC gel	Type II	20 mg/L MB, 20 mL, 0.5 g/L catalyst, 400 W Xe lamp	∼94% in 3 h	0.062	bacterial cellulose matrix improves adsorption and structural stability	Qian et al.^38^
TiO_2_/Bi_3_O_4_Br	n–n heterojunction	10 mg/L MB, 100 mL, 0.4 g/L catalyst, 50 W LED lamp	∼94%	0.066	strong n–n interaction enhances charge lifetime and broadens spectral response	Salmanzadeh-Jamadi et al.^39^
TiO_2_@In_2_S_3_	S-scheme	10 mg/L MB, 100 mL, 0.5 g/L catalyst, visible light	100% in 60 min	0.077	chrysanthemum-like morphology enhances active surface and redox synergy	Hilal Al Dhamri et al.^40^
WO_3_/TiO_2_	Type II	10 mg/L MB, 50 mL, 50 mg catalyst, 350 W Xe lamp	∼88% in 2 h	–	hollow structure promotes light trapping and fast interfacial migration	Wang et al.^41^
CQDs/ZnO	Type II	1 mM MB, 50 mL, 0.005 g catalyst, visible light	100% in 60 min	–	green-synthesized CQDs enhance conductivity and visible-light harvesting	Shalini et al.^42^
CuCo_2_S_4_/ZnO	Type II	0.01 mM MB, 250 mL, 0.4 g/L catalyst, 50 W LED lamp	∼93%	0.0178	active radicals from persulfate boost oxidative degradation efficiency	Habibi et al.^43^
CuO/ZnO	Type II	10 mg/L MB, 40 mL, 10 mg catalyst, 500 W Xe lamp	∼99% in 150 min	0.060	efficient visible-light response and charge separation through CuO loading	Shanmugam et al.^44^
MgFe_2_O_4_/ZnO/Perlite	Type II	10 mg/L MB, 50 mL, 0.5 g catalyst, 5 W LED	∼90%	0.055	perlite support improves stability; magnetic recovery supports reusability	Shoaib et al.^45^
V–ZnO/rGO	Type II	10 mg/L MB, 50 mL, 0.01 g catalyst, sunlight	94% in 4 h	0.0137	V-doping and rGO improve light absorption and magnetic separation	Zabihi and Monadi^46^
ZnO/MgFe_2_O_4_	Type II	10 mg/L MB, 75 mL, 0.3 g catalyst, 500 W halogen lamp	∼71%	0.0193	magnetic recovery; improved photocatalysis via enhanced separation	Shoaib et al.^47^
ZnO–ZnTe	S-scheme	8 mg/L MB, 50 mL, 30 mg catalyst, 150 W Xe lamp	∼91% in 2 h	0.0188	ZnTe narrows band gap; solar harvesting with good photocatalytic response	Raza et al.^48^
Bi_2_WO_6_/WO_3_	Type II	20 mg/L MB, 50 mL, 0.05 g catalyst, 30 W LED lamp	∼94% in 6 h	0.0141	staggered band alignment enhances charge migration and light utilization	Belousov et al.^49^
Bi_2_WO_6_/TiS_2_	Z-scheme	20 mg/L MB, 100 mL, 10 mg catalyst, visible light	91% in 70 min	0.049	Strong Z-scheme pathway boosts e^−^/h^+^ pair separation and dye degradation	Tanveer et al.^50^
MoS_2_/Bi_2_WO_6_	p–n heterojunction	20 mg/L MB, 50 mL, 10 mg catalyst, white 1 W LED lamp	97% in 60 min	0.0518	expanded MoS_2_ layers assist visible light capture and interfacial migration	Lai et al.^51^
CuO/BiVO_4_	p–n heterojunction	20 mg/L MB, 100 mL, 50 mg catalyst, 450 Xe lamp	99 in 2 h	0.1234	rapid degradation and dual functionality for Cr(VI) and MB removal	Chopade et al.^52^
BiVO_4_/g-C_3_N_4_	Z-scheme	10 mg/L MB, 200 mL, 0.2 g catalyst, 26 W natural light	97% in 3 h	0.0166	synergistic dye and antibiotic degradation through a visible-light-induced pathway	Tran et al.^53^
Bi_2_MoO_6_–Ag_2_MoO_4_	type II	10 mg/L of MB, 100 mL, 3 mg catalyst, visible light	∼92% in 160 min	0.0178	antibacterial and photocatalytic dual functionality, excellent stability	Balasurya et al.^54^
Bi_2_MoO_6_/LaFeO_3_	Z-scheme p–n	10 mg/L MB, 100 mL, 0.5 g/L catalyst, visible light	∼87% in 120 min	–	immobilized nanosheets on nanofibers enhance contact and photoreaction	Xu et al.^55^
β-Bi_2_O_3_/g-C_3_N_4_	Z-scheme	10 mg/L MB, 100 mL, 0.06 g/L catalyst, visible light	∼98% in 90 min	0.0407	Bi_2_O_3_ nanorods enhance redox potential and charge carrier separation	Nagar and Basu^56^
BiOBr–BiOI	type II	5 mg/L MB, 100 mL, 1 g/L catalyst, 150 W halogen lamp	97% in 120 min	–	plasma treatment improves surface reactivity and junction efficiency	Goudarzi et al.^57^
BiOBr/BiOCl–TiO_2_ QDs	Type II n-p-p	1 × 10^−5^ M MB, 250 mL, 0.1 g catalyst, 50 W LED	∼98% in 40 min	–	decoration of TiO_2_ QDs boosts light harvesting and electron mobility	Shoja et al.^58^
BiOBr/ZnO/BiOI	p–n–p heterojunction	1 × 10^−5^ M MB, 250 mL, 0.1 g catalyst, 50 W LED	∼94% in 60 min	0.066	triple junction ensures multichannel charge migration and stability	Zarezadeh et al.^59^
CdS/ZnWO_4_/ZnS	dual Z-scheme	10 mg/L MB, 100 mL, 50 mg catalyst, 300 W Xe lamp	∼100% in 50 min	0.1487	0D/1D/0D interface maximizes surface activity and radical generation	Zhang et al.^60^
MoS_2_/CdS/Bi_2_MoO_6_	Z-scheme	10 mg/L MB, 50 mL, 30 mg catalyst, 300 W Xe lamp	∼96% in 120 min	0.0256	ternary interface balances redox potentials for stable degradation cycle	Wang et al.^61^
Fe_3_O_4_/TiO_2_/g-C_3_N_4_	Z-scheme	10 mg/L MB, 100 mL, 50 mg catalyst, 300 W Xe lamp	95% in 80 min	0.03437	magnetically recyclable, effective against dyes and heavy metals	Madima et al.^62^
MIL-101(Fe)/Ce/g-C_3_N_4_	type II	15 mg/L MB, 50 mL, 15 mg catalyst, 300 W Xe lamp	∼88% in 75 min	0.0332	MOF-based structure increases surface area and light harvesting ability	Zhang et al.^63^
Mn_0__.__6_Zn_0__.__4_Fe_2_O_4_@Zn_0__.__9_Mn_0__.__1_O	type II	20 mg/L MB, 100 mL, 0.1 g catalyst, 300 Xe lamp	∼100% in 90 min	0.065	waste-derived photocatalyst, magnetic recovery, eco-friendly synthesis	Huang et al.^64^
Mn-Bi_2_WO_6_–GO/MoS_2_	Z-scheme	10 mg/L MB, 100 mL, 50 mg catalyst, visible light	∼99% in 60 min	0.0866	Mn doping and MoS_2_ nanosheets enable visible activity and fast carrier separation	Tahir et al.^65^
MnMgPO_4_/C_3_N_4_	S-scheme	10 mg/L MB, 100 mL, 0.5 g/L catalyst, 300 W Xe lamp	∼97% in 40 min	0.0959	bimetallic phosphate boosts redox properties and hydrogen co-generation	Cheng et al.^66^
MnTiO_3_–TiO_2_/C nanofiber	type II	10 mg/L MB, 100 mL, 0.6 g/L catalyst, visible light	∼91% in 120 min	0.0068	electrospun nanofibers offer large active area and stable performance	Yousef et al.^67^
Mo–Bi_2_WO_6_/WO_3_/Biochar	S-scheme	10 mg/L MB, 100 mL, 50 mg catalyst, 500 W halogen lamp	∼88% in 30 min	0.0281	biochar matrix enhances dispersion and electron mobility, stable hybrid	Rana et al.^68^
MoS_2_/ZnFe_2_O_4_	Z-scheme	10 mg/L MB, 100 mL, 10 mg catalyst, 160 W W-Hg lamp	∼92% in 160 min	0.015	improved charge dynamics, narrow bandgap pairing for visible light activation	Laxmiputra et al.^69^
MnMoO_4_·H_2_O/g-C_3_N_4_ tubes	S-scheme	10 mg/L MB, 200 mL, 0.1 g catalyst, 50 W LED lamp	∼94% in 90 min	0.062	tubular g-C_3_N_4_ provides fast electron channels, strong redox retention	Lahootifar et al.^70^
g-C_3_N_4_/Ti_3_C_2_T_x_/Ag_3_PO_4_	Z-scheme	10 mg/L MB, 60 mL, 60 mg catalyst, visible light	100% in 60 min	–	MXene enhances electron transfer, Ag_3_PO_4_ extends visible light response	Liao et al.^71^
g-C_3_N_4_/ZnO-W/Co_x_	type II	10 mg/L MB, 100 mL, 0.05 g/L catalyst, visible light	∼90% in 90 min	0.037	Co_x_ species improve charge separation, ZnO-W improves surface interaction	Malik et al.^72^
g-C_3_N_4(p)_/AgCl	type II	2.5 mg/L MB, 100 mL, 0.1 g catalyst, visible light	∼91% in 3 h	0.029	photogenerated Cl· radicals assist degradation, g-C_3_N_4_ enhances stability	Jiménez-Rangel et al.^73^
g-C_3_N_4_/Nanodiamond	type II	30 mg/L MB, 25 mL, 25 mg catalyst, visible light	∼89% in 120 min	0.0104	nanodiamond improves dispersion and charge mobility, no toxic metals	Kublik et al.^74^
Gd-BiFeO_3_/g-C_3_N_4_	type II	10 mg/L MB, 100 mL, 50 mg catalyst, solar light	93% in 100 min	0.0129	Gd doping enhances solar response and charge carrier mobility	Shakir^75^
GO/g-C_3_N_4_	S-scheme	30 mg/L MB, 100 mL, 30 mg catalyst, 500 W W-halogen	∼82% in 60 min	0.050	2D–2D heterojunction boosts electron transport and adsorption synergy	Su et al.^76^
GO–CNT/AgI	type II	1 mg/L MB, 100 mL, 1 g/L catalyst, 500 W W-halogen	∼91% in 90 min	0.3796	GO–CNT backbone increases conductivity, AgI facilitates visible-light activity	Shanthini et al.^77^
CuO/CeO_2_–ZrO_2_	p–n heterojunction	10 mg/L MB, 100 mL, 0.05 g/L catalyst, visible light	98% in 3 h	–	stable p–n junction facilitates improved separation and visible light response	Noman et al.^78^
F-g-C_3_N_4_/In_2_O_3_	type II	10 mg/L MB, 100 mL, 0.3 g/L catalyst, visible light	97% in 60 min	0.1784	fluorine doping improves charge transfer and pollutant degradation in real water	Chopade et al.^79^
Fe_3_O_4_/BiVO_4_/CuS	Z-scheme	10 mg/L MB, 100 mL, 0.5 g/L catalyst, 300 W Xe lamp	∼934% in 90 min	0.0274	magnetic separation, simultaneous Cr(VI) reduction and dye removal	Xu et al.^80^
FeOOH QDs/CuFe_2_O_4_	S-scheme	10 mg/L MB, 60 mL, 50 mg catalyst, 300 W Xe lamp	∼92% in 3 h	0.01047	chemically bonded interface improves charge transfer and broad-spectrum degradation	Shi et al.^81^
GACN/NiO/Ni_3_(BO_3_)_2_	dual Z-scheme	20 mg/L MB, 50 mL, 20 mg catalyst, 300 W Xe lamp	∼97% in 60 min	0.0482	strong redox capacity and high photostability for dual contaminant degradation	Guo et al.^82^
g-C_3_N_4_/BiVO_4_	Z-scheme	20 mg/L MB, 100 mL, 0.2 g/L catalyst, 300 W Xe lamp	∼99% in 100 min	0.066	nitrogen vacancies facilitate fast charge migration and prolonged carrier lifetime	Zhong et al.^83^
g-C_3_N_4_/CaTiO_3_/CQDs	Z-scheme	10 mg/L MB, 50 mL, 10 mg catalyst, 500 W Xe lamp	∼80% in 2 h	0.0117	CQDs act as electron shuttles, enhancing visible light absorption and conductivity	Zhao et al.^84^
g-C_3_N_4_/Co/ZnO	S-scheme	15 mg/L MB, 50 mL, 20 mg catalyst, 300 W Xe lamp	∼96% in 90 min	–	Co doping introduces mid-gap states and boosts aerobic photocatalytic degradation	Leelavathi et al.^85^
g-C_3_N_4_/ferrite/biochar	type II	20 mg/L MB, 50 mL, 25 mg catalyst, 500 W Xe lamp	∼97% in 2 h	0.0283	biochar and ferrite contribute synergistically to charge separation and adsorption	Sun et al.^86^
Ag/g-C_3_N_4_/LaFeO_3_	Z-scheme	10 mg/L MB, 60 mL, 20 mg catalyst, 300 W Xe lamp	∼99% in 2 h	–	plasmonic Ag enhances light harvesting and interfacial charge migration	Zhang et al.^87^
g-C_3_N_4_/CdS	type II	20 mg/L MB, 200 mL, 0.2 g catalyst, visible light	95% in 100 min	–	enhanced charge separation and visible light harvesting via CdS coupling	Shenoy et al.^88^
CaTiO_3_/g-C_3_N_4_	type II	20 mg/L MB, 50 mL, 10 mg catalyst, 500 W Xe lamp	100% in 2 h	0.0163	facile synthesis, g-C_3_N_4_ improves photocatalytic performance and stability	Zhao et al.^89^
CdS/Bi_4_Ti_3_O_12_	Z-scheme	5 mg/L MB, 100 mL, 1 g/L catalyst, 300 W Xe lamp	∼96% in 50 min	0.0659	core-shell architecture supports strong interfacial contact and charge migration	Cheng et al.^90^
ZnO/SiO_2_–cellulose hydrogel	type II	20 mg/L MB, 50 mL, 0.5 g/L catalyst, 300 W UV light	95% in 2 h	0.0254	*in situ* structured hydrogel matrix offers high reusability and sustainability	Ren et al.^91^
CeO_2_/g-C_3_N_4_	Z-scheme	20 mg/L MB, 100 mL, 20 mg catalyst, 250 W Xe lamp	∼100% in 150 min	0.0171	molten salt synthesis enables high crystallinity and redox potential preservation	Ning et al.^92^
CeO_2_@PDA/BiOBr	Z-scheme	30 mg/L MB, 60 mL, 30 mg catalyst, 300 W Xe lamp	100% in 30 min	0.1017	PDA mediates electron transfer; Z-scheme boosts visible-light activity	Zhang et al.^93^
Chitosan–TiO_2_/ZnO	Type II	30 mg/L MB, 125 mL, 0.4 g/L catalyst, sunlight	∼91% in 5 h	0.0079	biopolymer-assisted nanocomposite enhances dispersion and recovery	Rahman et al.^94^
Co–NiS/S–g-C_3_N_4_	Z-scheme	10 mg/L MB, 100 mL, 0.1 g catalyst, visible light	∼98% in 32 min	–	1D/2D hybrid promotes spatial charge separation and pathogen disinfection	Abubshait et al.^95^
Co_x_Ni_1-x_TiO_3_/CdS	Z-scheme	10 mg/L MB, 50 mL, 0.3 g catalyst, 500 W Xe lamp	99% in 75 min	–	visible-light-driven dual-component Z-scheme with strong photocatalytic redox	Reza Amani-Ghadim et al.^96^
Cs_3_Bi_2_Cl_9_/g-C_3_N_4_	Z-scheme	10 mg/L MB, 100 mL, 0.1 g catalyst, 55 W Xe lamp	∼99% in 150 min	0.0291	direct Z-scheme heterojunction with rapid charge carrier dynamics	Derikvand et al.^97^
Ag/ZrO_2_/TCN	S-scheme	20 mg/L MB, 100 mL, 0.8 g/L catalyst, 30 W LED lamp	∼97% in 2 h	–	enhanced interfacial charge transfer, ML-aided design, strong redox potential	Shiekhmohammadi et al.^98^
Ag@ZnO@Ti_3_C_2_	Z-scheme	10 mg/L MB, 50 mL, 50 mg catalyst, 300 W Xe lamp	∼91% in 50 min	0.0473	Ti_3_C_2_ MXene improves electron mobility, Ag plasmonic effect boosts light response	Liu et al.^99^
Ag_2_O@CRA	type II	20 mg/L MB, 100 mL, 1 g/L catalyst, visible light	91% in 60 min	–	recyclable photocatalyst, core-shell interface aids separation	Adday et al.^100^
Ag_2_WO_4_/CoWO_4_	type II	10 mg/L MB, 100 mL, 0.3 g/L catalyst, 500 W Xe lamp	∼94%	0.013	synergistic photocatalysis and antibacterial activity, suppressed recombination	Elgorban et al.^101^
Al_2_O_3_/CdO	type II	25 mg/L MB, 20 mL, 10 mg catalyst, white light	∼97% in 2 h	0.018	porous Al_2_O_3_ improves surface area, structural stability and photocatalytic efficiency	Janani et al.^102^
Bi/CdS/TiO_2_	S-scheme	10 mg/L MB, 30 mL, 0.5 g/L catalyst, 300 W Xe lamp	100% in 2 h	0.049	nanotube morphology supports charge transfer and hydrogen evolution	Wang et al.^103^
Bi/S-Bi_2_O_2_CO_3_	plasmonic heterojunction	10 mg/L MB, 60 mL, 0.25 g/L catalyst, visible light	∼80% in 2 h	0.017	sulfur activation enhances electron-hole mobility, strong visible response	Luo et al.^104^
BiVO_4_:Er^3+^,Yb^3+^/BiOCl	S-scheme	20 mg/L MB, 120 mL, 0.3 g/L catalyst, full-spectrum	∼98% in 12 h	0.012	rare-earth doping extends light range, improves redox potential	Liu et al.^105^
BiVO_4_/g-C_3_N_4_/rGO	Z-scheme	50 μM MB, 200 mL, 0.5 g/L catalyst, UVA light	∼96% in 2 h	0.075	rGO enhances electron transport, dual function for pollutant and microbial control	Akechatree et al.^106^

## Conceptual framework

### Photocatalytic fundamentals and semiconductor band theory

**Figure 2:**
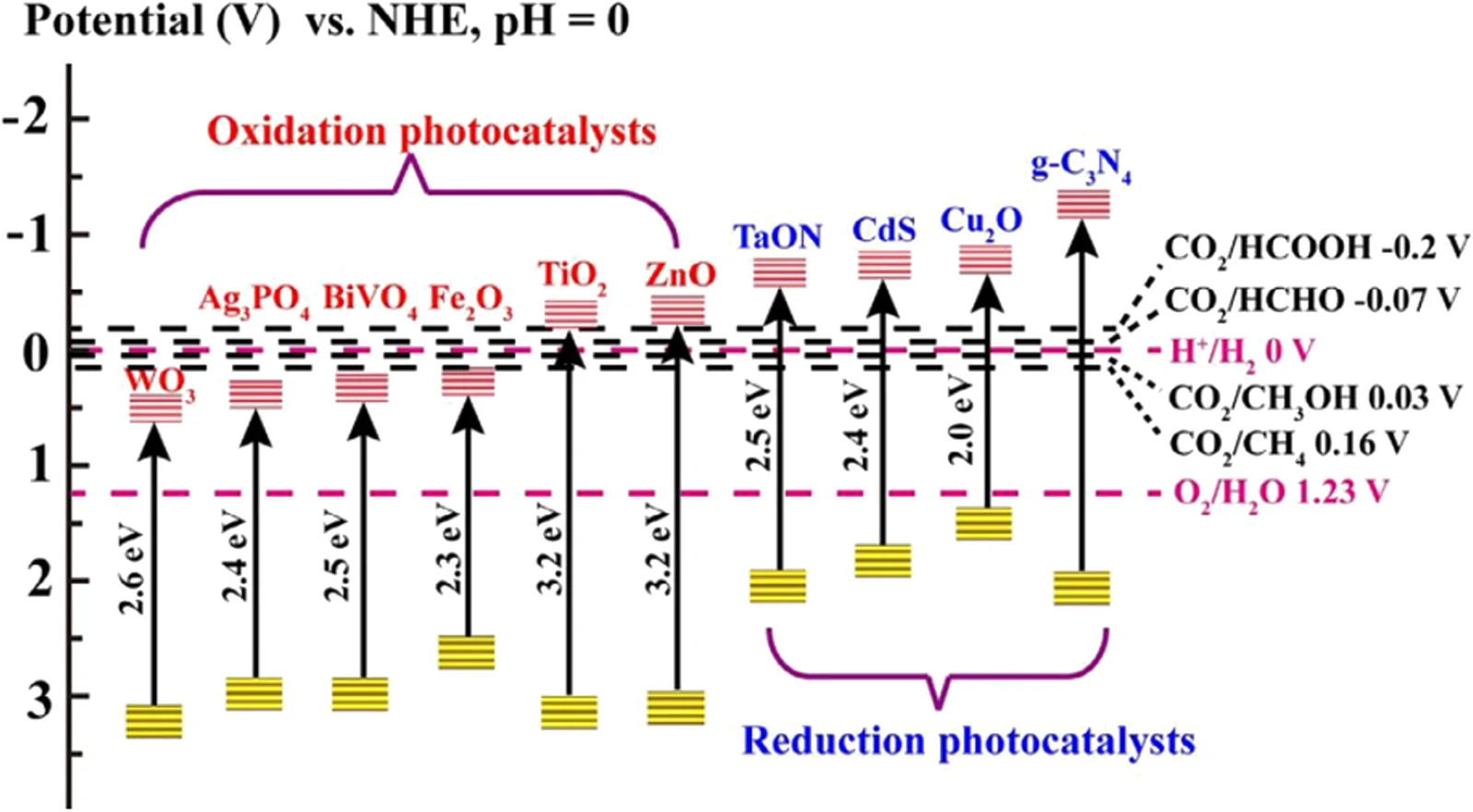
Band structures of some photocatalysts used in this review (reproduced from Xu et al.^107^ Copyright 2018 Elsevier)

Photocatalysis is an advanced oxidation process that uses light to activate semiconductor materials, thereby generating reactive species capable of degrading organic pollutants. When a semiconductor photocatalyst absorbs photons with energy equal to or greater than its bandgap, electrons are excited from the valence band (VB) to the conduction band (CB), leaving behind positively charged holes. These charge carriers interact with water and oxygen to form hydroxyl radicals and superoxide anions that can oxidize a wide range of organic compounds into harmless end products such as CO_2_ and H_2_O. Despite its theoretical efficiency and environmental compatibility, photocatalysis in practical applications has been limited by several factors. Most conventional photocatalysts, including TiO_2_ and ZnO, possess wide bandgaps (about 3.2 eV, Figure 2).^107^ This means they can only be activated by ultraviolet light. Since UV light accounts for only about 4% of the solar spectrum, this limits the use of solar energy in large-scale photocatalytic applications. Furthermore, the photogenerated electron-hole pairs tend to recombine quickly, thereby reducing the availability of reactive species and limiting overall photocatalytic performance.^12^^,^^108^ To overcome these challenges, researchers have developed heterostructured photocatalysts by combining two or more semiconductor materials with aligned energy bands. These heterostructures enhance charge separation and extend the spectral response into the visible light region, thereby improving photocatalytic efficiency. However, the degree to which these performance gains translate into genuine mechanistic advancement varies across junction types.

The performance of any heterojunction is governed by two semiconductor properties. These include the CB and VB edge potentials of the individual components, and their Fermi level positions before contact. Although related, these parameters serve different functions and should not be treated as equivalent when evaluating heterojunction mechanisms.

The CB and VB edge potentials of a semiconductor are usually estimated from optical and electrochemical data using the Mulliken electronegativity approach. The CB minimum is given by:

(Equation 1) $$E_{\text{CB}}=\chi -E_{e}-½E_{g}$$

Where: χ is the geometric mean of the atomic electronegativities of the constituent elements (the absolute electronegativity of the semiconductor)

E_e_ is the energy of free electrons on the hydrogen scale (approximately 4.5 eV), and

E_g_ is the optical bandgap measured from the Tauc plot.

The VB maximum is calculated as:

(Equation 2) $$E_{\text{VB}}=E_{\text{CB}}+E_{g}$$

These CB and VB positions define the thermodynamic window within which a semiconductor can drive reductive and oxidative photocatalytic reactions. A CB more negative than −0.33 V vs. NHE is necessary for superoxide radical generation via one-electron O_2_ reduction, while a VB more positive than +1.99 V vs. NHE is required for ·OH generation via direct water oxidation.^107^^,^^109^

On the other hand, the Fermi level represents the electrochemical potential of electrons at thermal equilibrium. In n-type semiconductors, the Fermi level is located close to the CB because donor states or oxygen vacancies increase the electron concentration. In p-type semiconductors, it lies nearer the VB due to the presence of acceptor states that lower the equilibrium electron density.^110^ Differences in Fermi level position between two semiconductors provide the thermodynamic driving force for charge redistribution when they are brought into contact.

Upon contact, electrons migrate spontaneously from the semiconductor with the higher Fermi level to that with the lower Fermi level until equilibrium is reached. This process creates a space-charge region and an internal electric field (IEF) directed from the higher-to the lower-Fermi-level material. The strength of this field depends on the initial Fermi level difference and can be estimated from flat-band potentials obtained by Mott–Schottky analysis or from work-function measurements using ultraviolet photoelectron spectroscopy (UPS) and Kelvin probe force microscopy (KPFM).^103^^,^^110^ The function of this interfacial electric field, and the direction in which it routes photogenerated charge carriers, differs across junction types and is the main variable distinguishing the mechanistic performance of heterojunction architectures.

### Heterojunction types and charge-transfer pathways

#### Type I heterojunctions

**Figure 3:**
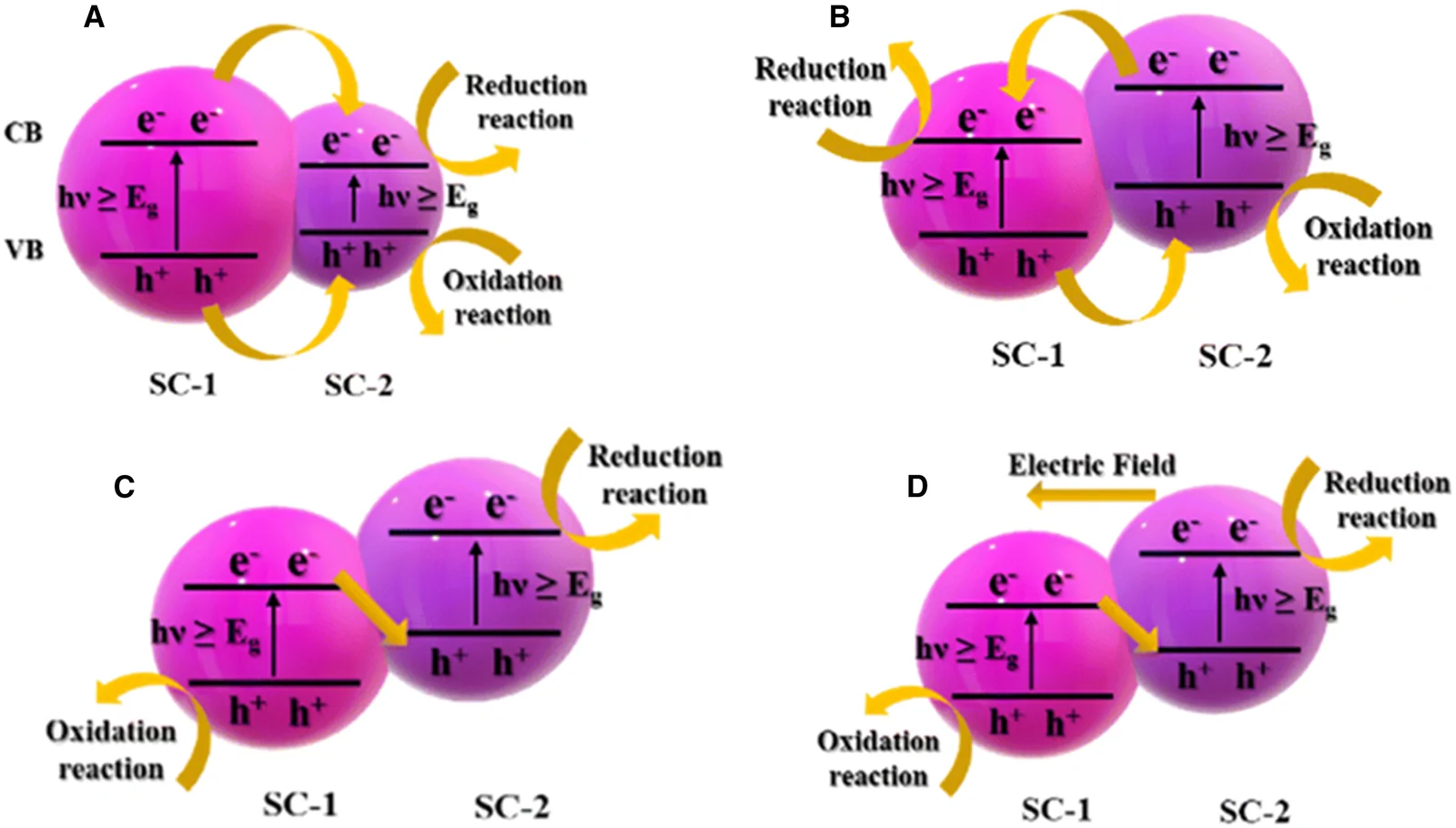
Schematic Comparison of Type I, Type II, Z-Scheme, and S-Scheme Heterojunctions Schematic illustration of (A) Type I, (B) Type II, (C) Z-scheme, and (D) S-scheme heterojunctions between semiconductors SC-1 and SC-2 (reproduced from Balapure et al.^111^ under CC BY-NC 3.0 license).

A Type I heterojunction (charge transfer mechanism illustrated in Figure 3A), also referred to as a straddling gap junction, involves the complete encapsulation of the conduction and VBs of one semiconductor within those of another. This configuration is mechanistically the least productive of all heterojunction architectures. Because both the CB minimum and VB maximum of the composite are localized on the same semiconductor component, photogenerated electrons and holes accumulate in the same material rather than separating across the interface. The consequence is that the junction provides no spatial driving force for charge segregation, and recombination rates in Type I systems are often comparable to, or only marginally lower than, those of the individual components. This fundamental limitation explains why Type I configurations are rarely the deliberate design target in photocatalytic literature and are more commonly encountered as an outcome of poorly matched band alignment.

#### Type II heterojunctions

A more functionally viable configuration is the Type II or staggered band alignment heterojunction (Figure 3B). In this case, the CB minimum of one semiconductor is positioned at a lower energy than that of the second, while the VB maximum is at a higher energy, creating a thermodynamic gradient that drives electrons toward the lower CB and holes toward the higher VB.^107^^,^^109^ This spatial separation of charge carriers suppresses recombination and, in principle, sustains both oxidative and reductive photocatalytic pathways simultaneously. Type II systems are by far the most frequently reported heterojunction class in the MB photocatalysis literature, but their prevalence reflects the accessibility of the design logic rather than its superiority. However, the spatial separation of carriers is achieved at the cost of reducing the redox potentials of both the retained electron and the retained hole. Electrons transferred to the lower CB possess a diminished reduction potential, while holes accumulated in the higher VB carry a reduced oxidation driving force.

The interfacial electric field in Type II systems promotes electron transfer to the lower CB and hole transfer to the higher VB, thereby improving charge separation but reducing the redox power of the remaining carriers.^107^^,^^109^ This limitation has direct mechanistic consequences. In the WO_3_/TiO_2_ Type II system reported by Wang et al.,^41^ electrons transferred from the TiO_2_ CB to the WO_3_ CB (∼0.0 V vs. NHE) retain only marginal driving force for O_2_ reduction (E^0^ = −0.33 V vs. NHE). This energetic cost and its performance consequence are analyzed in detail for the WO_3_/TiO_2_ system (Section TiO2-Based Heterostructures). The Bi_2_WO_6_/WO_3_ Type II system reported by Belousov et al.^49^ similarly required 6 h to achieve 94.1% MB removal. Across the Type II systems compiled in Table 1 of this review, including CuCo_2_S_4_/ZnO,^43^ MgFe_2_O_4_/ZnO,^45^ and Bi_2_MoO_6_-Ag_2_MoO_4_,^54^ this performance ceiling is a consistent feature that is rarely acknowledged by the original authors. The field would benefit from routine reporting of the redox potentials of the transferred carriers alongside standard photocurrent and PL data, as this would make the energetic cost of Type II operation explicit rather than concealed within performance comparisons.

#### Z-scheme heterojunctions

The Z-scheme heterojunction (charge transfer mechanism illustrated in Figure 3C), inspired by the natural photosynthetic Z-scheme in green plants, was developed to address the redox penalty inherent to Type II operation. In this configuration, photogenerated electrons in the CB of one semiconductor (the oxidation photocatalyst, OP) recombine selectively with holes in the VB of another (the reduction photocatalyst, RP). This selective recombination eliminates the weaker carriers from both components while preserving the most energetic electrons in the RP CB and the most oxidizing holes in the OP VB, thereby maintaining high redox power while improving charge separation efficiency.^107^^,^^109^ In mediator-assisted Z-scheme systems, this recombination is facilitated by a solid electron mediator, usually a noble metal such as Ag, deposited at the interface.

However, a limitation of mediator-free direct Z-scheme systems must be acknowledged. Without an internal thermodynamic force that dictates which carrier recombines with which, the assignment of the Z-scheme pathway in many literature reports rests on indirect scavenger experiments and band position calculations, without direct experimental confirmation of charge transfer direction. In the TiO_2_-CdS/bacterial cellulose composite reported by Qian et al.,^38^ which achieved 94.47% MB removal within 210 min, the Z-scheme assignment relies on performance comparisons and band position arguments rather than spectroscopic verification. The CdS/ZnWO_4_/ZnS dual Z-scheme system reported by Zhang et al.,^60^ despite achieving ∼100% MB removal in 50 min in a 0D/1D/0D morphological architecture, similarly relies on scavenger experiments and band structure calculations rather than time-resolved spectroscopic evidence. For a system in which three pairwise charge transfer events must operate cooperatively, the probability that one pathway dominates without being independently verified is non-trivial, and the exceptional performance may reflect the favorable interfacial morphology as much as the proposed dual Z-scheme mechanism.

#### S-scheme heterojunctions

A mechanistically unique configuration is the step-scheme (S-scheme) heterojunction. Although the S-scheme shares a superficial resemblance with the Z-scheme (both involve the selective recombination of less energetic charge carriers while preserving the most reactive ones), the two differ in their physical driving force. In an S-scheme system, the two component semiconductors are an OP with a lower Fermi level and an RP with a higher Fermi level. Upon intimate interfacial contact, spontaneous electron transfer from the RP to the OP occurs until the Fermi levels equilibrate, generating a built-in IEF directed from the RP to the OP at the heterojunction interface (Figure 3D). This charge redistribution simultaneously produces band bending: the conduction and VBs of the RP bend upward near the interface, while those of the OP bend downward. Under illumination, this IEF acts as the explicit thermodynamic driving force for selective recombination of photogenerated electrons in the OP CB with holes in the RP VB. This leaves behind the high-energy electrons in the RP CB and the strongly oxidizing holes in the OP VB for productive redox chemistry. The resulting charge transfer pathway traces an “S”-shaped path on an energy band diagram, giving the heterojunction its name.

Unlike Type II systems, the S-scheme simultaneously preserves strong reduction potential in the RP and strong oxidation potential in the OP, making it well-suited to the degradation of recalcitrant pollutants such as MB that require both high ·OH and high ·O_2_^−^ yields. Unlike mediator-free Z-scheme systems, the IEF and band bending in the S-scheme provide a physically grounded driving force for directional charge transfer that does not depend on the spatial proximity of oppositely charged carriers.

An important consequence of Fermi level equilibration in S-scheme systems is band bending. Electron migration across the interface alters the electrostatic potential within the space-charge region, causing upward band bending in the RP and downward band bending in the OP near the interface. This effect strengthens selective carrier recombination and is a defining characteristic of true S-scheme behavior. S-scheme heterojunctions rely on Fermi level equilibration involving an RP with a higher Fermi level and an OP with a lower Fermi level. The resulting electric field extends from the RP to the OP and facilitates selective recombination between low-energy electrons in the OP CB and low-energy holes in the RP VB. As a result, highly reducing electrons remain in the RP CB, while strongly oxidizing holes remain in the OP VB.^103^ This configuration preserves both reduction and oxidation potentials, thereby enabling efficient generation of ·O_2_^−^ and ·OH radicals. Consequently, S-scheme systems such as TiO_2_@In_2_S_3_ and MnMgPO_4_/g-C_3_N_4_ exhibit superior photocatalytic performance compared with analogous Type II structures.^40^^,^^66^

#### p–n heterojunctions

**Figure 4:**
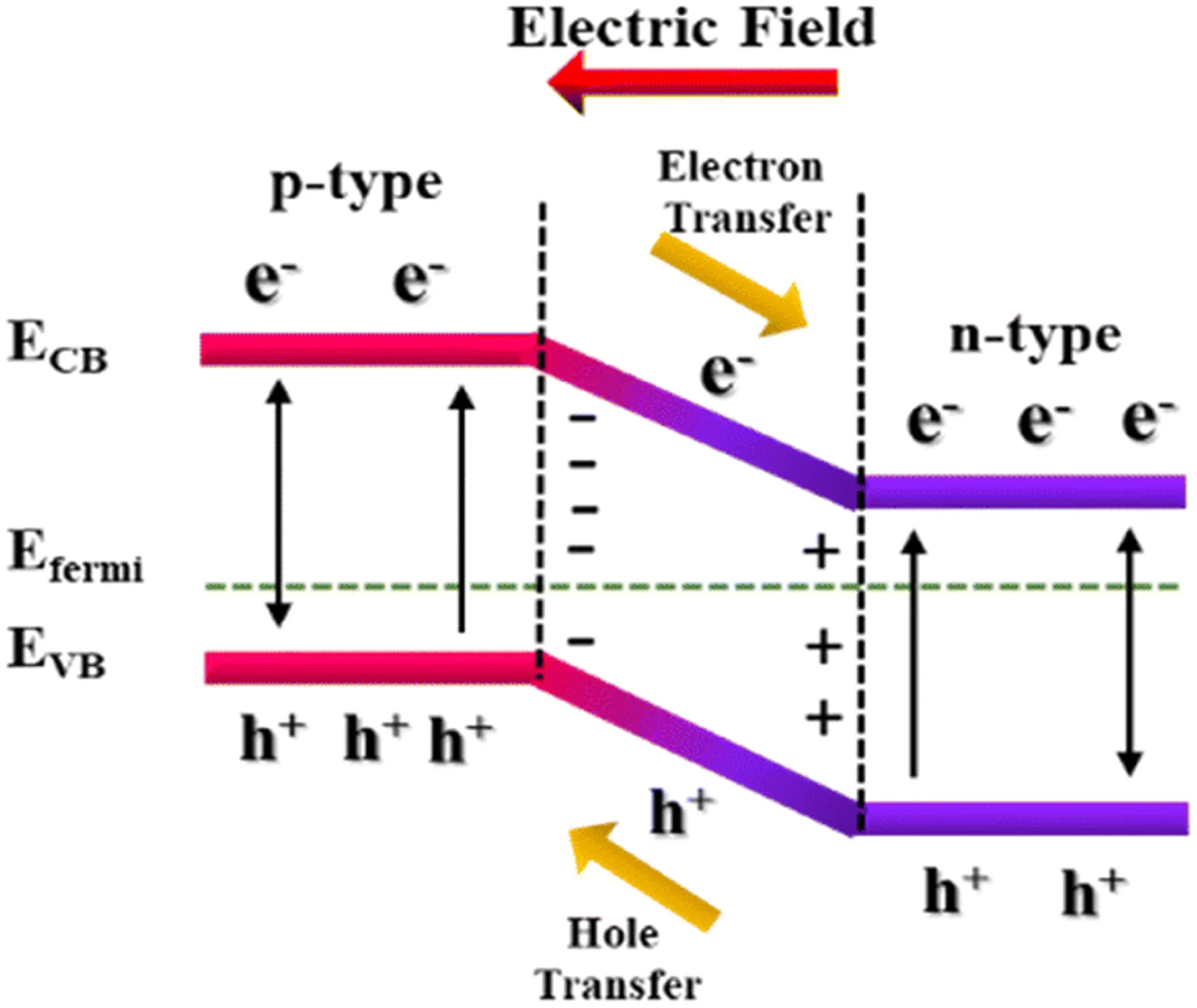
Schematic illustration of the band structure and the charge recombination processes in a p-n heterojunction (reproduced from Balapure et al.^111^ under CC BY-NC 3.0 license)

Another widely studied configuration is the p–n heterojunction, which forms when a p-type semiconductor is paired with an n-type semiconductor.^110^ The difference in charge carrier concentrations between the two components leads to spontaneous formation of an IEF at the junction interface, which drives electrons and holes in opposite directions, thereby enhancing charge separation and suppressing recombination (Figure 4). In p–n heterojunctions, this field arises from differences in carrier concentration between the p-type and n-type semiconductors and directs electrons toward the n-type region and holes toward the p-type region. This behavior has been verified in systems such as MoS_2_/Bi_2_WO_6_ and ZnO/CuO through Mott-Schottky measurements.^44^^,^^51^

The p-n junction is among the most mechanistically verifiable of all heterojunction types, because the n-type or p-type character of each component can be independently confirmed by Mott-Schottky analysis, and the direction of the IEF follows directly from well-established semiconductor contact physics. This verifiability distinguishes p-n systems from Z-scheme and S-scheme assignments that rest on indirect evidence. However, verification is not always rigorous in practice. The ZnO/CuO p-n system reported by Shanmugam et al.,^44^ in which p-type CuO nanorods (bandgap ∼1.2–1.5 eV) decorate n-type ZnO, achieving 98.5% MB degradation within 150 min under visible light, stands as one of the more robust mechanistic assignments in this review. This is because the semiconductor type of both components is independently well-established and the direction of carrier flow is thermodynamically consistent with the reported band positions. By contrast, the MoS_2_/Bi_2_WO_6_ p-n heterojunction reported by Lai et al.,^51^ while providing quantitative Mott-Schottky characterization and reporting a composite carrier density approximately 11 times higher than bare Bi_2_WO_6_, acknowledges that the flat-band potential measured on powder-based electrodes carries an uncertainty of 0.1–0.3 V. This margin, in some cases, is sufficient to alter the predicted band alignment and thus the proposed direction of charge flow. The CuO/BiVO_4_ p–n composite reported by Chopade et al.,^52^ which achieved 99% MB removal in 2 h alongside simultaneous Cr(VI) reduction, offers mechanistic elegance in using both carrier types in a single irradiation cycle, but similarly does not provide work function measurements or post-reaction surface analysis that would fully validate the proposed junction mechanism. These examples illustrate that the p–n framework, while conceptually the most accessible, still requires more rigorous quantitative support than most studies in this class currently provide.

#### Schottky junctions

Metal-semiconductor combinations form Schottky junctions, in which the metal component typically serves as an electron sink, collecting and trapping photogenerated electrons from the semiconductor CB and thereby reducing recombination at the semiconductor surface. These structures additionally provide localized surface plasmon resonance in noble metal systems, extending light absorption into the visible region and generating hot electrons that can be injected directly into the semiconductor CB.^112^^,^^113^ Schottky junctions also provide additional active sites for catalytic reactions at the metal-semiconductor interface. The Ag/g-C_3_N_4_/LaFeO_3_ Z-scheme system reported by Zhang et al.^87^ illustrates the roles that metallic components can play. Ag nanoparticles at the g-C_3_N_4_/LaFeO_3_ interface function simultaneously as a plasmonic light harvester and as a solid electron mediator facilitating Z-scheme charge transfer, and disentangling these two contributions is a persistent challenge that the study does not resolve. This ambiguity is a challenge in Schottky-functionalized systems, where the noble metal’s role as an electron mediator, a recombination site, or a plasmon sensitizer is rarely clarified by independent experimentation.

#### Van der Waals heterostructures

Recent interest has also focused on van der Waals heterostructures assembled from two-dimensional materials, including MoS_2_, g-C_3_N_4_, and graphene.^114^^,^^115^ These systems do not require lattice matching between components, thus offering a significant fabrication advantage over conventional heterostructures in which interfacial strain from lattice mismatch can introduce defect states that function as recombination centers. van der Waals heterostructures offer high interfacial contact area, excellent charge mobility, and electronically tunable properties arising from interlayer coupling. The MoS_2_/Bi_2_WO_6_ system described by Lai et al.,^51^ assembled using interlayer-expanded MoS_2_ nanosheets, illustrates the practical manifestation of this approach. The expanded interlayer spacing increases MB intercalation and improves interfacial contact with Bi_2_WO_6_, thereby contributing to 97% MB degradation in 40 min under white LED illumination. However, the tendency of two-dimensional materials to undergo restacking can progressively decrease the effective contact area and hinder MB transport to active interfacial sites. This limitation is rarely addressed in either the broader van der Waals literature or the systems reviewed here. Moreover, the lack of post-reaction electron microscopy verifying lamellar structural integrity after repeated cycles remains a significant gap in assessing practical durability claims. Jia et al. constructed a g-C_3_N_4_/BiLuWO_6_ composite and subjected it to combined theoretical and experimental characterization, which established the formation of a van der Waals heterojunction between the two components and identified a Z-scheme charge transfer pathway as the operative mechanism.^116^ Under the same irradiation conditions, the heterojunction delivered faster MB photodegradation than either constituent phase in isolation.

Among the heterojunction architectures discussed here, the p–n junction rests on the most firmly established semiconductor physics, while the S-scheme remains the most debated in terms of the experimental evidence required to confirm it. The challenge confronting the field is not the design of new material combinations, but the adoption of characterization standards commensurate with the mechanistic complexity being claimed.

### Characterization standards for mechanistic assignment

Most of the studies reviewed here assign heterojunction types (whether Type II, Z-scheme, S-scheme, or p-n) based on a standard toolkit of indirect characterization methods, including Mott–Schottky analysis, photoluminescence (PL) quenching, transient photocurrent measurements, electrochemical impedance spectroscopy (EIS), and free radical scavenger experiments. While these tools are informative and experimentally accessible, they are individually insufficient to establish a charge transfer pathway.

Mott–Schottky plots determine flat-band potentials and semiconductor type, but these values are sensitive to measurement frequency, electrolyte composition, and surface states, and they do not directly reveal what happens to carriers after junction formation. PL quenching confirms that recombination is suppressed in the composite, but cannot distinguish between Type II and Z-scheme/S-scheme pathways, since all three suppress PL relative to the single components. Scavenger experiments identify the dominant reactive species (·OH, ·O_2_^−^, h^+^) but cannot resolve the spatial origin of those species or the direction of charge flow.

The critical distinction between a Z-scheme and a Type II junction (including whether the oxidative power of the VB holes is preserved or sacrificed) requires direct evidence from techniques such as *in situ* X-ray photoelectron spectroscopy (XPS), electron paramagnetic resonance (EPR) spin trapping under illumination, or femtosecond transient absorption spectroscopy. For S-scheme systems specifically, the S-scheme is differentiated experimentally through *in situ* XPS under illumination, which reveals binding energy shifts consistent with the predicted band bending direction; EPR, which confirms simultaneous generation of both ·OH and ·O_2_^−^; and KPFM, which directly visualizes the surface potential redistribution and spatial resolution of the IEF.^41^ Evidence for band bending is usually obtained through *in situ* XPS, which detects illumination-induced shifts in core-level binding energies, or through KPFM, which directly maps surface potential gradients across the junction. However, these advanced characterizations have been applied to only a limited number of the MB photocatalytic systems reviewed in this work. Consequently, many reported S-scheme assignments rely primarily on calculated band structures and indirect evidence rather than direct observation of Fermi level-induced band bending.

The evidentiary gap is not confined to S-scheme systems. The TiO_2_@In_2_S_3_ S-scheme composite reported by Hilal Al Dhamri et al.,^40^ which achieved 100% MB removal within 60 min with degradation rates 6.5 times faster than pure TiO_2_, assigns the S-scheme mechanism on the basis of band edge calculations and radical trapping data, without *in situ* EPR or operando XPS confirmation of interfacial charge flow direction. Similarly, the MnMgPO_4_/g-C_3_N_4_ S-scheme system reported by Cheng et al.,^66^ which achieved ∼97% MB removal in 40 min with a high apparent rate constant of 0.0959 min^−1^, and the FeOOH QDs/CuFe_2_O_4_ composite reported by Shi et al.,^81^ rely on the same indirect toolkit. While the performance data for these systems are compelling, the mechanistic assignments remain consistent with but not proven by the available evidence. A significant number of recent papers assign the S-scheme label based solely on staggered band alignment combined with scavenger experiments suggesting simultaneous ·OH and ·O_2_^−^ participation. The field would benefit greatly from adopting stricter experimental standards for S-scheme assignment, especially the *in situ* XPS and KPFM evidence that are diagnostic of band bending and IEF formation.

## Design strategies and photocatalytic performance in MB removal

### TiO_2_-based heterostructures

TiO_2_ has long attracted attention as a photocatalyst for wastewater treatment, owing to its strong oxidation potential, chemical stability, and affordability. Its wide bandgap (∼3.2 eV) restricts photoactivation to the ultraviolet region, and rapid recombination of photogenerated electron-hole pairs further curtails efficiency. To address these limitations, researchers have coupled TiO_2_ with narrow-bandgap semiconductors, noble metals, or carbonaceous materials to construct heterostructures with enhanced visible-light absorption and improved charge-carrier dynamics. The charge-transfer behavior in these systems is governed by the CB and VB edge potentials of the paired semiconductors relative to TiO_2_ and by Fermi level alignment prior to contact.

**Figure 5:**
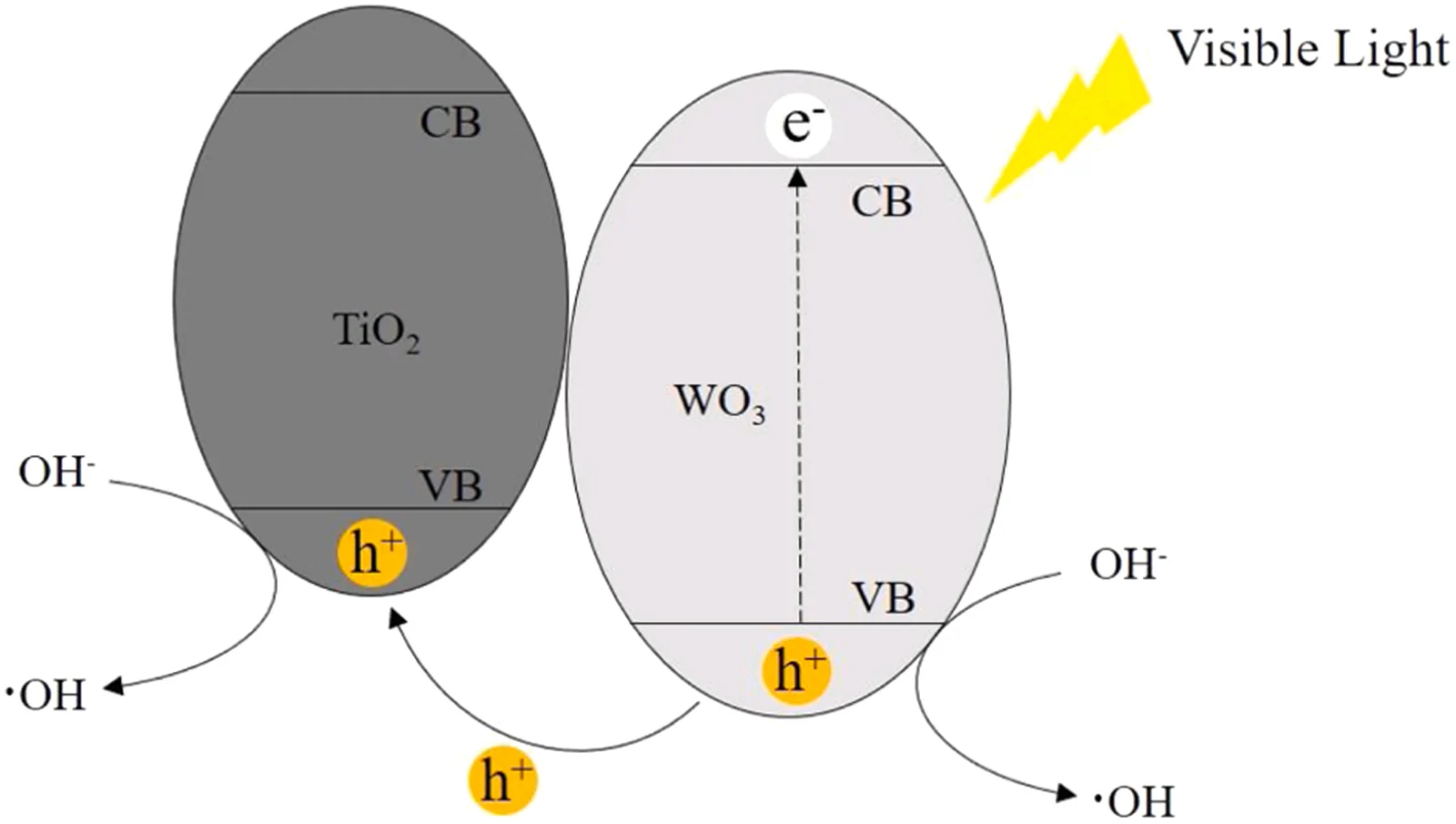
Schematic illustration of the photodegradation mechanism of WO_3_/TiO_2_ heterojunction photocatalyst (reproduced with permission from Wang et al.^41^ Copyright 2021 Elsevier)

Wang et al. fabricated a WO_3_/TiO_2_ heterostructured photocatalyst with a hollow spherical morphology via hydrothermal synthesis followed by calcination.^41^ The optimized 10 wt % WO_3_ composition achieved 87.8% MB degradation within 150 min under visible light, compared to 40.7% for pure TiO_2_. The improvement was attributed to Type II heterojunction formation, with staggered band alignment driving electrons from the TiO_2_ CB to that of WO_3_, supported by PL quenching and photocurrent enhancement. The staggered band alignment positions the WO_3_ CB below that of TiO_2_, making interfacial electron transfer thermodynamically favorable. However, this transfer carries a direct energetic penalty that the authors do not address. Electrons arriving at the WO_3_ CB (∼0.0 V vs. NHE) retain only marginal driving force for one-electron O_2_ reduction, whose thermodynamic threshold lies at −0.33 V vs. NHE. This shortfall constrains the rate of ·O_2_^−^ generation and is the mechanistic origin of the system’s performance ceiling of 87.8% MB removal after 150 min. This example illustrates a structural limitation of Type II operation. Spatial charge separation is achieved at the cost of reducing the redox potentials of the transferred carriers, and when the receiving CB falls below the O_2_/·O_2_^−^ reduction potential, superoxide generation becomes thermodynamically unfavorable regardless of how efficiently charge separation occurs. The photodegradation mechanism of the photocatalyst is illustrated in Figure 5.

Hilal Al Dhamri et al. developed a TiO_2_@In_2_S_3_ S-scheme composite via a facile hydrothermal route, achieving a bandgap of 1.88 eV (substantially narrowed from bare TiO_2_ (3.18 eV)), reflecting effective visible-light sensitization by β-In_2_S_3_ acting as the reduction photocatalyst.^40^ The optimized composite achieved 100% MB removal within 60 min, with degradation rates 6.5 and 3.8 times faster than pure TiO_2_ and β-In_2_S_3_, respectively. The proposed S-scheme mechanism invokes selective recombination of CB electrons from TiO_2_ with VB holes from β-In_2_S_3_, preserving the more reductive electrons in β-In_2_S_3_ and the more oxidative holes in TiO_2_ for ROS generation. This assignment is consistent with the band positions of the two semiconductors and corroborated by scavenger experiments, though it rests on band edge calculations and radical trapping data rather than *in situ* EPR or operando XPS. Notably, the CB and VB edge potentials of each component are not independently reported, and their precise values require verification by Mott–Schottky analysis or UPS. Nonetheless, the dramatic performance enhancement over single-component systems clearly establishes the value of coupling TiO_2_ with narrow-bandgap sulfide partners. Together, these two systems reveal that mechanistic assignments are rarely accompanied by the energetic analysis needed to bridge charge-transfer claims and practical ROS-generation outcomes.

Salmanzadeh-Jamadi et al. developed a TiO_2_/Bi_3_O_4_Br n-n heterojunction via single-step hydrothermal synthesis, achieving a 65.8-fold enhancement in MB degradation rate relative to bare TiO_2_.^39^ The assignment of an n–n junction was supported by Mott–Schottky plots confirming the n-type character of both semiconductors, and the IEF at the interface was invoked to explain directional charge separation. PL, photocurrent, and EIS measurements confirmed improved charge separation in the composite. The performance enhancement is striking, and the n–n assignment is internally consistent. However, it is worth noting that the formation of an IEF at an n–n junction requires a significant difference in work function between the two materials. Where this difference is modest, the driving force for charge separation is weaker than in a conventional p–n junction, and the photocatalytic advantage of the heterojunction over a simple physical mixture of the two components cannot always be guaranteed. The authors do not report work function measurements or contact potential differences that would validate the field-driven mechanism, which somewhat weakens the mechanistic interpretation while leaving the performance data intact.

Qian et al. demonstrated a TiO_2_-CdS/bacterial cellulose composite prepared through a microwave-assisted solvothermal route, which achieved 94.47% MB removal within 210 min under visible light.^38^ The Z-scheme charge transfer mechanism was proposed, supported by the significant improvement over either single component. A notable practical feature of this system is the cellulose matrix, which promoted catalyst recovery and additionally sequestered leached Cd^2+^ ions, addressing a major toxicity concern associated with CdS-containing photocatalysts. However, from a mechanistic point of view, the Z-scheme assignment rests primarily on performance comparisons and band position arguments rather than direct spectroscopic evidence. CdS is known to undergo photocorrosion under illumination through oxidation of S^2−^ by VB holes; a process that would be suppressed in a true Z-scheme if those holes are consumed in the recombination step, but exacerbated in a Type II configuration where they accumulate on CdS. The retention of photocatalytic activity over five cycles is suggestive but not conclusive evidence of Z-scheme operation, since the cellulose matrix independently prevents dissolution through physical sequestration. Clarifying this distinction has direct implications for the long-term stability claims made for this class of materials.

The TiO_2_-based heterostructure literature demonstrates convincing performance improvements across different semiconductor pairings. The most consistent design principle is that coupling TiO_2_ with a narrow-bandgap visible-light absorber (In_2_S_3_, CdS, WO_3_) extends the spectral response and suppresses recombination. The mechanistic basis for this improvement (whether Type II, Z-scheme, or S-scheme) is less established, and future studies in this family would benefit from including time-resolved PL decay measurements and EPR spin-trapping under illumination as standard characterization components.

### ZnO-based heterostructures

ZnO offers a complementary platform to TiO_2_. It offers high electron mobility and strong oxidation potential, but suffers from the same wide bandgap limitation (∼3.2 eV) and additional susceptibility to photocorrosion under prolonged illumination. Charge-transfer behavior in ZnO-based heterostructures is governed by the CB and VB edge potentials of ZnO and its paired semiconductor, as well as the Fermi level difference that drives band bending upon contact. ZnO is n-type, placing its Fermi level close to its CB; pairing it with a p-type semiconductor whose Fermi level lies near its VB generates a built-in electric field at the junction interface that directs electrons toward ZnO and holes in the reverse direction.

Shanmugam et al. reported ZnO/CuO heterostructured nanorods synthesized via a hydrothermal-calcination route, achieving 98.5% MB degradation within 150 min under visible light, compared to significantly lower performance for bare ZnO.^44^ The enhanced activity was attributed to a p–n heterojunction between p-type CuO (∼1.2–1.5 eV bandgap) and n-type ZnO, with the IEF driving electrons from the CuO CB to ZnO and holes in the reverse direction. This mechanistic assignment is among the more robust in the ZnO literature: the p-type character of CuO and n-type character of ZnO are well-established and independently verifiable, and the resulting superoxide generation from electrons accumulated at the ZnO CB (alongside direct oxidation by holes concentrated in the CuO VB) provides a coherent mechanistic picture. That said, CuO is susceptible to photoreduction to Cu^0^ under strongly reductive conditions, and post-reaction XPS monitoring of Cu valence state would be a valuable addition to validate the long-term stability claim.

Shoaib et al. incorporated magnetic MgFe_2_O_4_ into ZnO through a sol-gel route, achieving 71.0% MB degradation under visible light alongside magnetic catalyst recovery.^47^ The relatively modest efficiency (below the >90% routinely achieved by more sophisticated ZnO heterostructures) suggests suboptimal energetic alignment at the MgFe_2_O_4_/ZnO junction. The charge separation mechanism was described as CB electron transfer from MgFe_2_O_4_ to ZnO, supported by PL quenching, but this assignment must be scrutinized. The CB and VB edge potentials of MgFe_2_O_4_ are sensitive to stoichiometry and synthetic conditions and are not reported with sufficient precision to validate the proposed electron transfer direction or the Fermi level offset driving the claimed interfacial electric field. UPS or VB XPS measurements would substantially strengthen the mechanistic interpretation, even as the magnetic recovery feature retains practical value.

**Figure 6:**
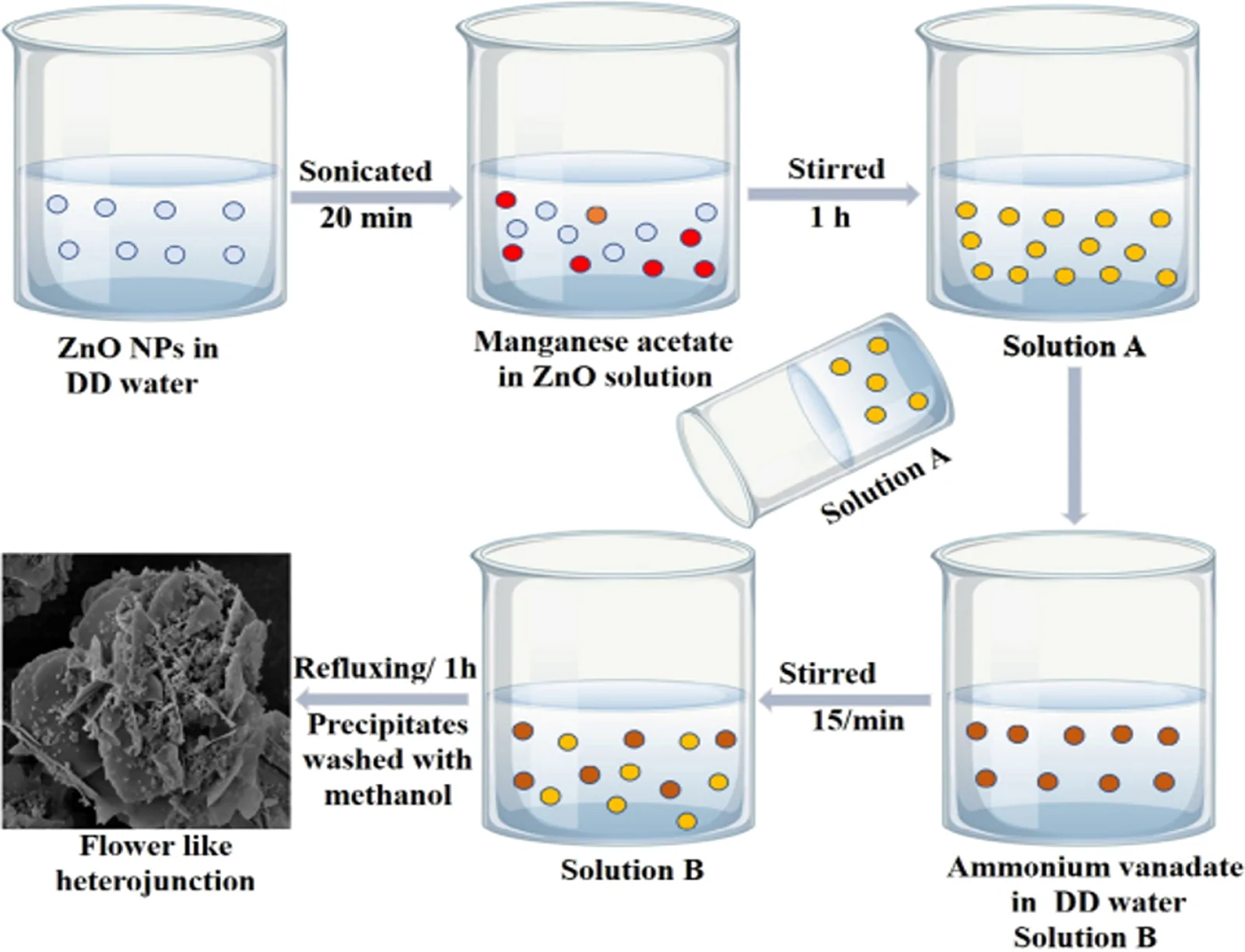
Illustration of the synthesis of flower-like ZnO/MnV_2_O_6_ heterojunction (reproduced with permission from Kaushal et al.^117^ Copyright 2021 Elsevier)

Kaushal et al. constructed a 3D flower-like ZnO/MnV_2_O_6_ architecture (synthesis scheme illustrated in Figure 6) exploiting combined adsorption-photocatalysis functionality.^117^ The dual mode is practically advantageous, as surface pre-concentration of MB through adsorption can increase local pollutant availability at active sites and enhance apparent degradation rates. However, reported degradation efficiencies may conflate true mineralization with adsorptive removal unless TOC measurements accompany UV-vis absorbance monitoring.

Habibi et al. extended the ZnO platform by introducing CuCo_2_S_4_ as a co-catalyst to activate persulfate under visible light, achieving MB degradation 3.56 times faster than pure ZnO.^43^ The synergy between photocatalysis and persulfate-derived sulfate radical generation is a well-established strategy in advanced oxidation, and this system effectively combines both routes. The mechanistic description (electron transfer from CuCo_2_S_4_ to ZnO, with holes retained in CuCo_2_S_4_) is consistent with a Type II or possibly Z-scheme configuration, but the authors do not provide definitive evidence to distinguish between them. More importantly, when persulfate is present, the dominant oxidative species shifts from hydroxyl radical to sulfate radical (E^o^ = 2.5–3.1 V vs. NHE), which has different selectivity and pH dependence. The mechanistic discussion does not disentangle the contributions of photocatalytic ROS generation from persulfate activation, which would be necessary to rationally optimize either pathway independently.

Raza et al. reported ZnO-ZnTe hierarchical superstructures achieving approximately 91% MB removal within 2 h under solar irradiation.^48^ The Type II heterojunction was proposed, with CB electron transfer from ZnTe to ZnO and hole migration in the reverse direction. The use of actual solar irradiation (rather than simulated light from a xenon lamp) is a noteworthy practical advantage that is underrepresented in this field. However, solar irradiance is variable and not reported in standardized units in this study, making quantitative comparison with lamp-based systems problematic. The ZnTe component is also notable in that it narrows the effective bandgap of the composite and contributes to visible-light absorption, but its stability under prolonged solar illumination (a concern for many telluride-based systems due to potential Te^4+^ leaching) is not evaluated.

The ZnO-based heterostructure literature illustrates a productive diversity of junction types and functional features. The p–n heterojunction with CuO stands out as mechanistically the most rigorously supported. A recurring weakness is the insufficient attention to photocatalyst stability under the conditions most relevant to real applications, including extended illumination, circumneutral to alkaline pH, and the presence of natural organic matter that competes for reactive species.

### Bismuth-based heterostructures

Bismuth-based semiconductors, including Bi_2_WO_6_, BiVO_4_, Bi_2_MoO_6_, and bismuth oxyhalides (BiOX, X = Cl, Br, I), are among the most active visible-light photocatalysts known, owing to their layered crystal structures, IEFs from [Bi_2_O_2_]^2+^ layers, and narrow bandgaps tunable across the visible spectrum. Their heterostructures have been productive for MB degradation but also exhibit some of the most contested mechanistic assignments in the field. The diversity of junction types observed across this material family reflects the tunability of CB and VB edge potentials within bismuth semiconductors and the Fermi level differences that arise when they are paired with complementary materials.

Belousov et al. developed a Bi_2_WO_6_/WO_3_ Type II heterojunction through hydrothermal synthesis, achieving 94.1% MB removal in 6 h under visible light.^49^ The Type II assignment was supported by band position calculations and the measured optical properties of each component. However, the 6-h irradiation time compares unfavorably with Z-scheme and p-n systems in the same material family that achieve comparable efficiencies in 40–90 min. This kinetic deficit is mechanistically attributable to the Type II configuration itself: electrons transferred to the WO_3_ CB accumulate at approximately 0.0 to +0.5 V vs. NHE, providing at best marginal reducing power relative to the O_2_ reduction threshold (E^o^ = −0.33 V vs. NHE), which limits superoxide radical generation and constrains overall degradation rate. This energetic penalty of Type II operation is an important mechanistic consideration that spatial charge separation alone does not offset.

In the MoS_2_/Bi_2_WO_6_ p-n system, Mott-Schottky analysis confirmed the p-type character of MoS_2_ and n-type character of Bi_2_WO_6_. This established a Fermi level offset that generates an IEF at contact.^51^ However, flat-band potentials measured on powder electrodes carry an uncertainty of 0.1–0.3 V, and this limitation applies broadly to Mott–Schottky-based mechanistic assignments across bismuth heterostructures.

Tanveer et al. assembled a Bi_2_WO_6_/TiS_2_ Z-scheme heterojunction via solvothermal synthesis, achieving 98% MB removal within 70 min^50^ This study provides more direct mechanistic evidence than many in the field. PL quenching confirmed reduced recombination, transient photocurrent measurements demonstrated enhanced charge separation, and the Z-scheme assignment is supported by the energetic argument that selective recombination of less-reactive Bi_2_WO_6_ CB electrons with TiS_2_ VB holes preserves the strongest reductant (TiS_2_ CB) and oxidant (Bi_2_WO_6_ VB) for degradation. This is more rigorous than most studies. The remaining gap is the absence of EPR spin-trapping data showing the specific radical species generated, and of operando measurements confirming that the recombination pathway indeed proceeds as proposed under realistic reaction conditions rather than in the absence of the pollutant substrate.

Lai et al. constructed a MoS_2_/Bi_2_WO_6_ p-n heterojunction through interlayer-expanded MoS_2_, achieving 97% MB degradation in 40 min under white LED illumination.^51^ The p-n junction assignment was supported by a V-shaped Mott–Schottky plot, confirming the coexistence of p-type (MoS_2_) and n-type (Bi_2_WO_6_) semiconductors in the composite, and the reported charge carrier density of 4.85 × 10^16^ cm^−1^ in the composite (approximately 11 times higher than bare Bi_2_WO_6_) provides a quantitative measure of the enhanced carrier availability arising from junction formation. This level of quantitative mechanistic characterization is notably stronger than the narrative descriptions typical of other studies discussed here. A remaining weakness is that the Mott–Schottky characterization was conducted on powder-based electrodes, subject to the same flat-band potential uncertainty of 0.1–0.3 V noted above, which in some cases is sufficient to alter the predicted direction of charge flow.

Chopade et al. prepared a CuO/BiVO_4_ p-n composite by chemical bath deposition for combined MB degradation and Cr(VI) reduction.^52^ The simultaneous dual-function capability (oxidative dye degradation and reductive heavy metal removal) is mechanistically elegant, as it leverages both types of charge carriers in a single irradiation cycle. This represents a practical advance that single-function systems cannot achieve. The mechanism was supported by band position calculations and scavenger experiments. As with the other p–n systems reviewed, work function measurements and post-reaction surface analysis to confirm the chemical integrity of both CuO and BiVO_4_ under the strongly reductive conditions required for Cr(VI) reduction would add important mechanistic depth.

The bismuth-based heterostructure literature is the richest in terms of mechanistic diversity, spanning Type II, Z-scheme, direct Z-scheme, and p–n configurations, and contains some of the most impressive performance data in this review. The mechanistic quality is also more variable. Tanveer et al. and Lai et al. provide notably stronger evidence than most, while several others rest their mechanism entirely on indirect measurements. A field-wide move toward standardized mechanistic reporting (including, at minimum, quantitative EPR spin-trapping and time-resolved PL lifetime measurements) would substantially clarify the true operating mechanisms in this productive material family.

### Ternary heterostructures

Ternary heterostructured photocatalysts introduce a third semiconductor into the composite architecture, creating multiple heterojunctions within a single material and offering additional degrees of freedom in band alignment, charge transfer cascade engineering, and functional integration. The mechanistic complexity of ternary systems is correspondingly greater, and the challenges of unambiguously assigning charge transfer pathways are amplified.

Zarezadeh et al. synthesized a BiOBr/ZnO/BiOI p–n–p ternary heterostructure through a controlled solvothermal route, achieving 95% MB degradation within 50 min under visible light.^59^ The p–n–p architecture was designed to generate two consecutive IEFs, driving stepwise charge migration: electrons from BiOBr→ZnO→BiOI, holes in the reverse direction. This staggered charge relay is mechanistically appealing in principle, but in practice, the energy barriers at each successive junction must be correctly aligned for the cascade to operate as proposed. The study provides structural characterization and PL data consistent with the heterojunction formation but does not verify the band alignment at each interface independently. In multi-junction ternary systems, it is quite possible for charge to accumulate at intermediate interfaces rather than propagating across all three components, a scenario that is kinetically different from the idealized cascade and would not be detected by standard characterization. Techniques such as KPFM, which can map surface potential distributions at the nanoscale, would provide direct evidence of the proposed multi-junction charge relay.

Shoja et al. anchored BiOBr and BiOCl nanosheets onto TiO_2_ quantum dots to achieve 92% MB degradation within 60 min^58^ The charge transfer description; electrons from BiOBr/BiOCl transferring to TiO_2_ QDs, holes retained on the bismuth halide components, is consistent with a Type II cascade, given the more negative CB positions of the bismuth halides relative to TiO_2_. One important mechanistic issue not discussed in the paper concerns the effect of quantum confinement on the TiO_2_ QDs. At the nanoparticle dimensions used, quantum size effects can shift band positions by up to 0.3–0.5 eV relative to bulk values, potentially altering the energetics of the proposed charge transfer. Bulk-referenced band position values, commonly used in these calculations, may therefore overestimate the thermodynamic driving force for electron injection into TiO_2_ QDs.

Zhang et al. reported a CdS/ZnWO_4_/ZnS dual Z-scheme heterostructure with a 0D/1D/0D morphology, achieving 98% MB removal in 40 min^60^ The dual Z-scheme is one of the most complex mechanisms proposed in the MB photocatalysis literature, and the performance data are correspondingly impressive. The mechanistic evidence relies on radical scavenger experiments and band structure calculations, without direct validation by time-resolved spectroscopy. For a system of this complexity, where three pairwise charge transfer events must operate cooperatively, the probability of one pathway dominating over others is non-trivial. The exceptional performance may also reflect the favorable 0D/1D/0D morphology (specifically, the intimate contact between nanoparticles and nanorods that minimizes interfacial resistance (rather than the dual Z-scheme mechanism per se. Disentangling morphological and mechanistic contributions to performance is a challenge that this and similar papers do not fully address.

Wang et al. constructed a MoS_2_/CdS/Bi_2_MoO_6_ Z-scheme heterostructure achieving 96% MB degradation in 45 min^61^ The Z-scheme assignment rests on the argument that Bi_2_MoO_6_ CB electrons recombine with MoS_2_ VB holes, thereby preserving CdS CB electrons and MoS_2_ VB holes for redox reactions. As with CdS-containing systems discussed in Section TiO2-based heterostructures, the photocorrosion susceptibility of CdS in the absence of hole consumption is a persistent concern. The observed activity retention over multiple cycles is encouraging, but TOC measurements and post-reaction ICP-MS analysis for Cd^2+^ leaching would be necessary to confirm true stability rather than apparent stability due to re-adsorption.

Madima et al. prepared a Fe_3_O_4_/TiO_2_/g-C_3_N_4_ ternary composite achieving 95% MB degradation in 60 min with the practical advantage of magnetic recovery.^62^ The Z-scheme mechanism proposed (recombination of g-C_3_N_4_ CB electrons with TiO_2_ VB holes, preserving TiO_2_ CB electrons and g-C_3_N_4_ VB holes) is among the most commonly invoked in the TiO_2_/g-C_3_N_4_ literature. This is a well-studied pairing, and there is now a body of evidence from time-resolved spectroscopy in TiO_2_/g-C_3_N_4_ binary systems that supports a Z-scheme-like charge transfer pathway (though the mechanism is still debated in some configurations). Extending this understanding to the ternary Fe_3_O_4_-containing system is reasonable but not directly verified. The Fe_3_O_4_ component is magnetically useful but may also act as a recombination center for photogenerated carriers; an effect that the study acknowledges is mitigated by the spatial separation afforded by the ternary architecture, but which is not quantitatively assessed.

The ternary heterostructure section reveals a field of impressive performance achievements accompanied by mechanistic interpretations of increasing complexity that are not always matched by correspondingly rigorous experimental support. The most important methodological recommendation for future ternary heterostructure studies is the systematic application of component-selective photocatalytic experiments. These include testing binary sub-systems alongside the ternary to confirm that the performance increment arises from the third component’s mechanistic contribution and not from its effect on surface area, light scattering, or pollutant adsorption.

### Carbon-integrated supports

The integration of carbon-based materials (especially graphene and graphite) into photocatalytic systems has emerged as a recurring strategy for improving MB degradation efficiency, primarily through their capacity to suppress charge carrier recombination and extend visible-light absorption. While the performance gains reported across recent studies are significant, a critical assessment reveals that the mechanistic roles attributed to carbon components are frequently inferred rather than rigorously demonstrated, and the long-term stability of such composites under real-world conditions remains insufficiently interrogated.

Graphene has been deployed both as a functional co-catalyst and as a structural support in MB photodegradation systems. In the study by Ojeda et al.,^118^ LaFe_0.94_V_0.01_Mn_0.05_O_3_ (LFV) nanoparticles were deposited onto graphene-coated cotton fabric substrates, thereby yielding an ESP/LFV composite that achieved 96.3% MB degradation after only 10 min of solar irradiation. This is a marked improvement over the 91.1% degradation attained with bare LFV powder under identical conditions. The authors attributed this enhancement to the electron-trapping capability of graphene, which retarded electron-hole recombination and consequently promoted superoxide radical (·O_2_^−^) generation (identified as the predominant oxidizing species via scavenger experiments). While this mechanistic interpretation is consistent with established graphene photocatalysis literature, the study offers no time-resolved PL or transient absorption spectroscopy to directly quantify recombination kinetics. This gap limits the mechanistic conclusions to inference. Furthermore, the 10-min degradation timeline was demonstrated for a single MB concentration (20 ppm) in tap water, and the recyclability and structural integrity of the graphene-coated fabric under repeated solar exposure were not systematically reported. These omissions are consequential for any claim of practical applicability.

A more unconventional approach to carbon material utilization is presented by Valadez-Renteria et al.,^119^ who investigated graphite recovered from spent alkaline (Duracell) and lithium-ion (Huawei, Lenovo) batteries as standalone photocatalysts for MB degradation. Graphite from alkaline batteries (DBAA) achieved complete MB mineralization (30 mg/L) within 30 min under sunlight. This performance is substantially superior to graphite recovered from lithium-ion sources (LBH: 92–99.9% in 90 min; LBL: comparable range). The superior activity of DBAA graphite was mechanistically linked to its higher density of surface oxygen functional groups (identified by XPS as C=O and C–O species) and the presence of residual Zn and Mn oxide impurities from the battery chemistry, both of which generated additional surface defects confirmed by Raman spectroscopy. These defects functioned as electron-trapping centers that accelerated ROS generation, with TOC analysis confirming 92% mineralization for DBAA. This study is notable for its circular economy framing. Recycling toxic e-waste as a functional photocatalytic material addresses both resource recovery and water remediation simultaneously. Nevertheless, a critical concern arises from the leaching potential of residual heavy metals (Zn, Mn) into the treated water stream, especially given the demonstrated role of these impurity phases in photocatalytic activity. The authors do not present aqueous-phase metal analysis post-treatment, which is a significant oversight in the context of remediation safety. In addition, the graphite powders were subsequently immobilized on graphene-coated polyurethane sponge composites (SGr/DBAA, SGr/LBH, SGr/LBL), achieving MB degradation efficiencies above 96% under sunlight in 90 min. While immobilization aids practical recovery, the composite architecture introduces a secondary carbon interface (the graphene coating) whose independent contribution to photocatalytic performance is not deconvoluted from the graphite component.

In a parallel context, graphene was used as the primary electrode substrate in the study by Rosales et al.,^120^ where Ni-doped MnBi_2_O_4_ (MnNiBi) powders were deposited on graphene-printed flexible polyethylene electrodes. Although the primary focus of that work was dual-function energy storage and photocatalysis, the graphene component played a structural rather than photocatalytic role in the degradation experiments, thereby serving as a flexible conductive substrate rather than an active charge-transfer mediator in the photocatalytic cycle. Unlike the ESP/LFV system discussed above, the graphene here did not appear to contribute directly to MB degradation kinetics, and the reported 98% MB degradation under UV light was attributed to the oxygen vacancies introduced by Ni doping rather than to the carbon support.

These studies establish that carbon materials (whether in the form of pure graphene, functionalized graphite, or recycled battery-grade carbon) contribute meaningfully to MB photodegradation through electron-trapping, defect engineering, and substrate functionality. However, the field continues to lack rigorous mechanistic differentiation between carbon’s role as an electron shuttle, a light absorber, or an adsorption enhancer. Systematic studies employing *in situ* spectroelectrochemical characterization, combined with controlled leaching and long-term recyclability assessments, are necessary before carbon-integrated photocatalysts can be considered viable candidates for scalable water treatment applications.

## Structure-performance relationships

The textural properties and surface morphologies of photocatalytic materials are two aspects of the same structure-performance question. Pore type, size, and spatial organization govern molecular accessibility, adsorption pre-concentration, and ROS delivery at the solid-liquid interface, while surface morphology determines active site density, light scattering efficiency, interfacial contact area between heterojunction components, and the transport of ROS to the pollutant. Yet despite their recognized importance, pore characteristics are inconsistently reported across the literature, and the mechanistic interpretation of morphological observations remains frequently underdeveloped. A critical reading of the SEM, TEM, and BET evidence across material families also exposes a persistent disconnect between structural observation and mechanistic attribution that the field must address.

### TiO_2_-based systems

Among TiO_2_-based photocatalysts, mesoporous structures (pore diameter 2–50 nm) are the most thoroughly characterized pore type. The Fe_3_O_4_-SiO_2_-mesoTiO_2_ nanocomposite^121^ exhibits a BET surface area of 197.8 m_2_/g, a pore volume of 0.34 cm^3^/g, and a narrow pore diameter of 3.4 nm. N_2_ adsorption-desorption analysis confirmed the mesoporous nature of this composite, which achieved 97.52% MB photodegradation under UV–Vis irradiation alongside a meaningful pre-adsorption contribution of 23.32%. The 3.4 nm pore diameter is comparable to the molecular dimensions of MB (∼1.43 nm in its longest axis), suggesting that while MB can enter the mesopore network, pore confinement effects may restrict diffusion kinetics and influence the orientation of MB at the photocatalyst surface. Similarly, Juliya et al.^122^ synthesized mesoporous TiO_2_/RuO_2_ nanosystems via a sol-gel route using Pluronic P123 as a structure-directing agent, with N_2_ sorption confirming Type IV isotherms with H1-type hysteresis. The H1 hysteresis specifically indicates that pore connectivity is high and that capillary condensation occurs in a relatively uniform channel geometry, facilitating unobstructed MB diffusion and contact with photocatalytic sites.

In contrast, the porous TiO_2_ film prepared via the doctor’s blade method represents an architecturally distinct category. With TiO_2_ nanoparticles of ∼100 nm embedded in a 25 μm thick film, the resulting porosity is interparticulate and irregular in nature.^123^ Achieving 90% MB degradation across four reuse cycles, this system demonstrates that structural porosity, even without controlled pore geometry, can sustain reasonable performance. However, the absence of BET characterization in this study prevents any rigorous correlation between pore dimensions and photocatalytic output, which is a significant reporting gap that limits the mechanistic insight the data could otherwise offer.

**Figure 7:**
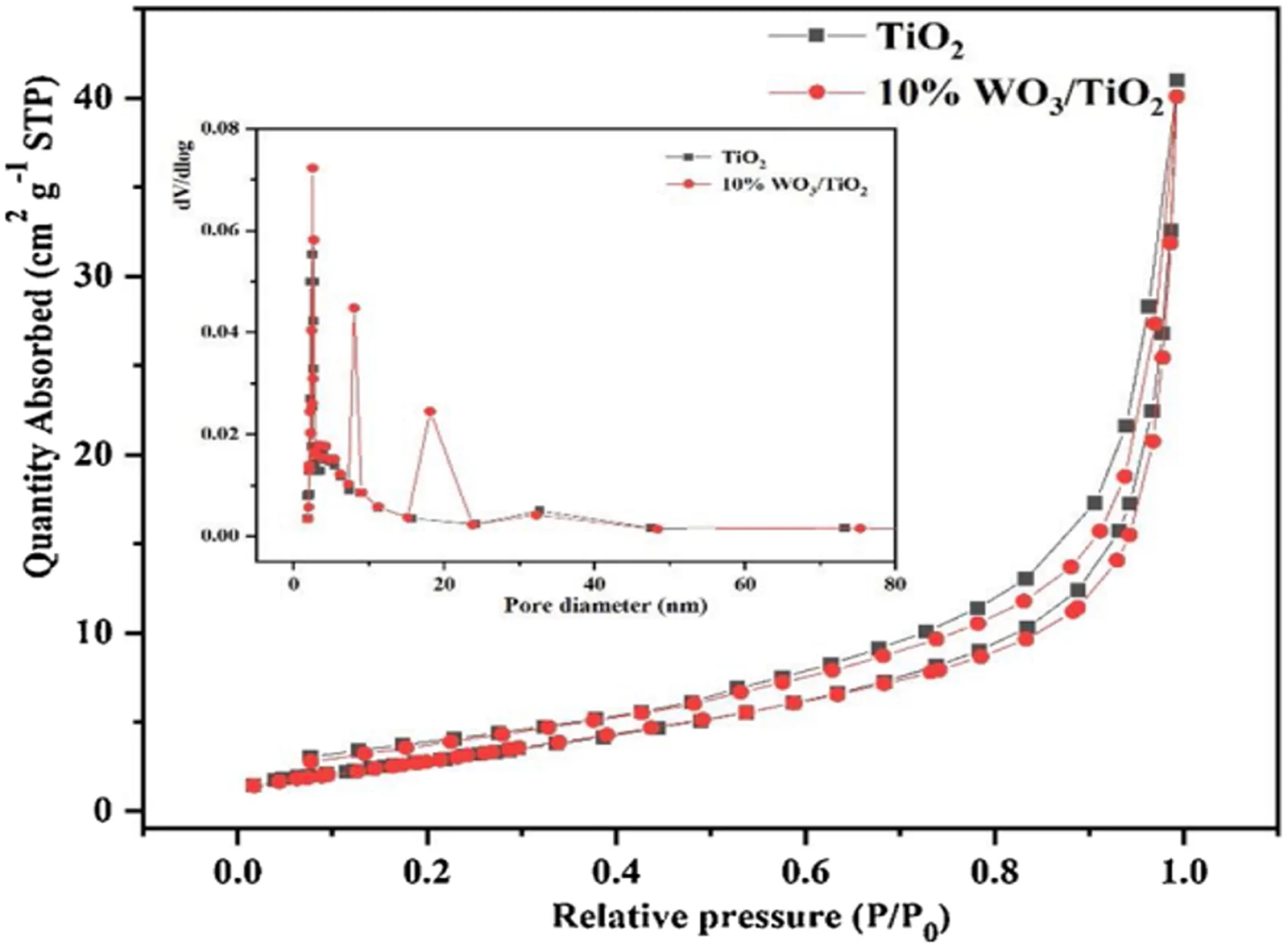
N_2_ adsorption-desorption isotherms and the corresponding pore size distribution profiles of TiO_2_ and 10% WO_3_/TiO_2_ samples (reproduced with permission from Wang et al.^41^ Copyright 2021 Elsevier)

The N_2_ adsorption-desorption isotherms and corresponding pore size distribution curves for pure TiO_2_ and the 10% WO_3_/TiO_2_ composite of the study by Wang et al.^41^ further illustrate these pore-performance relationships (Figure 7). Both materials exhibited Type IV isotherms characterized by a pronounced upturn at a relative pressure of approximately 0.9, consistent with mesoporous architectures undergoing capillary condensation. Pore size analysis revealed that the dominant pore diameters in both samples fell within the 2–10 nm range, with the composite showing a marginal increase in the contribution of pores around 20 nm relative to pure TiO_2_. This modest broadening of the pore size distribution is mechanistically relevant, as larger mesopores facilitate the diffusion of pollutant molecules into the interior surface of the material, thereby increasing the probability of productive contact between MB and active photocatalytic sites. BET surface area measurements yielded values of 11.4470 and 11.7505 m^2^/g for pure TiO_2_ and the 10% WO_3_/TiO_2_ composite, respectively. This indicates that WO_3_ incorporation produces only a negligible change in total surface area, and that the observed enhancement in photocatalytic performance cannot be attributed to surface area gains alone.

**Figure 8:**
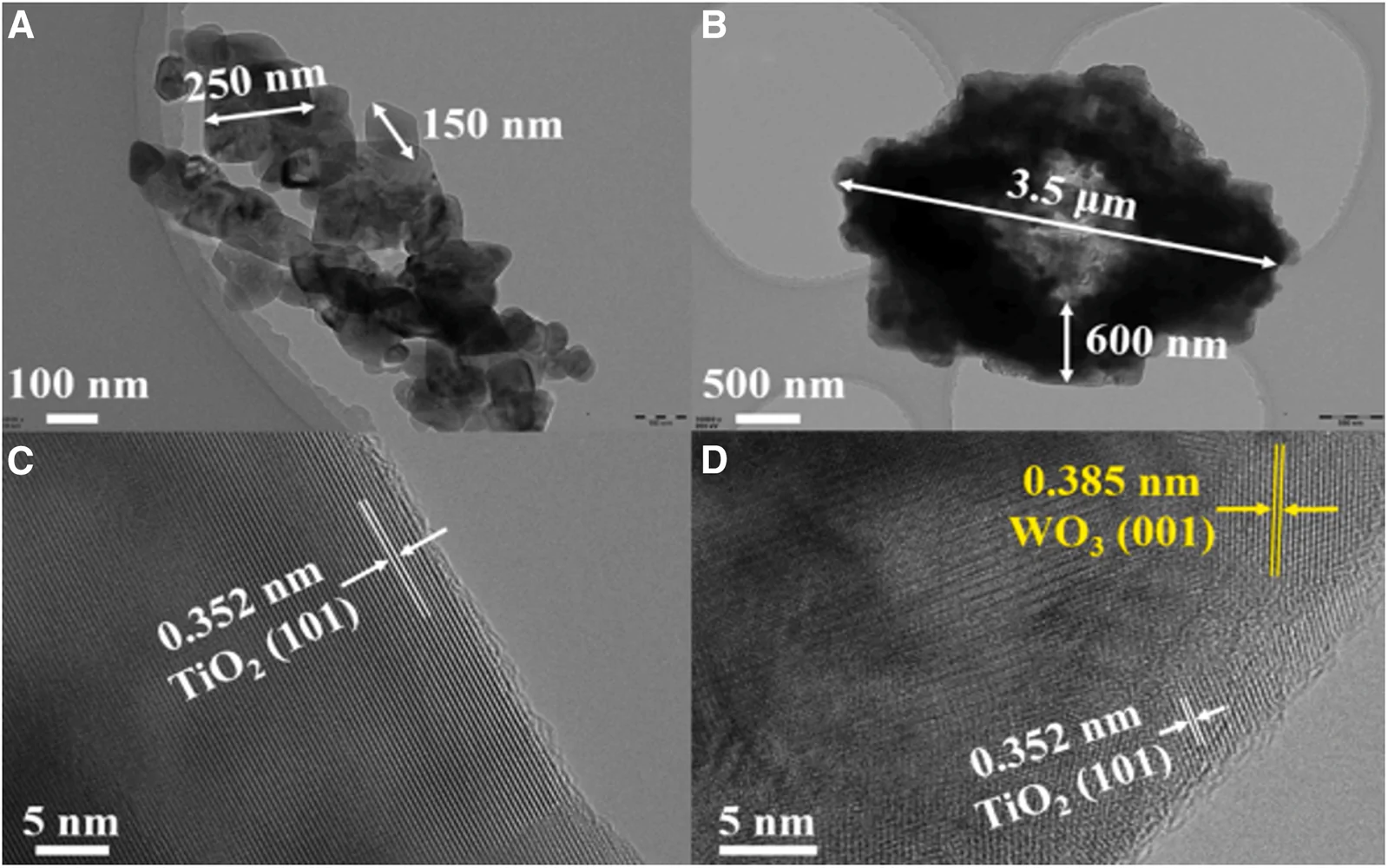
TEM and HRTEM Images of TiO2 and 10% WO3/TiO2 Composite TEM and HRTEM micrographs of (A and C) pure TiO_2_ and (B and D) the 10% WO_3_/TiO_2_ composite (reproduced with permission from Wang et al.^41^ Copyright 2021 Elsevier).

The morphological picture for TiO_2_-based composites is equally instructive. The WO_3_/TiO_2_ composite fabricated by Wang et al.^41^ exhibits a hollow spherical morphology achieved by hydrothermal synthesis followed by calcination. Hollow spheres are optically advantageous because multiple internal light reflections increase the effective photon path length through the catalytic material, thereby improving photon utilization relative to solid particles of equivalent diameter. The hollow architecture also reduces the diffusion distance for MB from the external solution to the active inner surface. Nevertheless, the study does not decouple the morphological contribution from the Type II junction mechanism in rationalizing the 87.8% MB degradation in 150 min, and the extent to which the hollow geometry specifically accelerates degradation is not experimentally isolated. The internal microstructure of pure TiO_2_ and the 10% WO_3_/TiO_2_ composite was further examined by TEM. As shown in Figure 8A, the samples comprised aggregated nanocrystals in the 100–200 nm size range, which assembled into the secondary hollow microsphere architecture observed in Figure 8B, consistent with the SEM morphology described earlier. High-resolution TEM (HRTEM) images in Figure 8C and Ed reveal well-defined lattice fringes indicative of high crystallinity. A d-spacing of 0.352 nm was measured and assigned to the (101) facets of anatase TiO_2_, while a fringe spacing of 0.385 nm was observed in Figure 8D corresponds to the (001) interplanar spacing of WO_3_. These lattice assignments are consistent with the XRD data, and together confirm the successful formation of a crystalline 10% WO_3_/TiO_2_ hollow microsphere composite with well-integrated interfacial structure.

In contrast, the TiO_2_@In_2_S_3_ S-scheme composite^40^ adopts a chrysanthemum-like morphology in which In_2_S_3_ petals radiate outward from a TiO_2_ core, maximizing the exposed heterojunction interfacial area. The chrysanthemum geometry also reduces interparticle aggregation. This failure mode is common in nanoparticle-based systems that compress accessible surface area and is rarely discussed quantitatively in the literature. The Bi/CdS/TiO_2_ nanotube morphology^103^ further demonstrates that one-dimensional tubular geometries facilitate unidirectional charge transport along the tube axis, suppressing lateral recombination and directing carriers toward terminal surface sites. Taken together across the TiO_2_ family, the pairing of mesoporous textural control with morphologically engineered geometries (hollow spheres, chrysanthemum architectures, and 1D nanotubes) generates compounding advantages in photon harvesting, MB pre-concentration, and charge transport that no single structural parameter delivers in isolation.

### ZnO-based systems

The ZnO family provides the most instructive direct comparison of how pore size magnitude alters MB removal outcomes. Zulfa et al.^124^ demonstrated that ZnO derived from a mixed-ligand ZIF-8 template (using 2-methylimidazole and 2-aminoterephthalic acid) possessed a substantially larger pore diameter of 79.14 nm compared to 13.81 nm for single-ligand-derived ZnO. This 6-fold increase in pore size translated into enhanced MB adsorption during the pre-photocatalytic phase, attributable to the reduced diffusional resistance within larger pore channels that allows MB molecules (which are bulky and planar) to access internal surface sites more readily. The mixed-ligand ZnO achieved ∼97.18% MB degradation in 90 min, with PL measurements confirming a lower charge carrier recombination rate relative to the single-ligand variant. The pore size of 79.14 nm places this material at the boundary between the mesoporous and macroporous regimes, suggesting that for large planar dye molecules like MB, transitioning toward larger pore diameters may be more beneficial than the highly ordered but dimensionally restrictive mesopores commonly reported for TiO_2_ systems. The Ru-ZnO@g-C_3_N_4_ nanocomposite^125^ also employed a mesoporous architecture confirmed by BET analysis, enabling visible-light-driven Z-scheme MB degradation, though the specific pore diameter and isotherm classification were not explicitly disclosed.

The morphology of ZnO-based systems reinforces these pore-level observations. The ZnO/CuO system prepared by Shanmugam et al. 2023) exhibits nanorod morphology confirmed by SEM, with CuO nanoparticles decorating the ZnO rod surfaces. The nanorod geometry is mechanistically meaningful for p–n junction systems. The high aspect ratio exposes predominantly non-polar facets along the rod length, which in ZnO are known to have different surface charge densities than the polar (0001) tip facets, potentially directing the spatial localization of photogenerated holes and electrons to distinct regions of the rod surface. This facet-selective charge segregation is an underexplored dimension of ZnO nanorod photocatalysis that has direct implications for which rod regions drive MB oxidation most efficiently. The ZnO/MnV_2_O_6_ composite^117^ adopts a three-dimensional flower-like hierarchical architecture, in which assemblies of nanosheet petals radiate from a central nucleus. This morphology generates both macroporous interflower voids and mesoporous intraflower spaces that concentrate MB near the catalytically active petal surfaces. The synergistic adsorption-photocatalysis dual functionality reported for this system is enabled by this hierarchical pore structure, though the failure to report TOC measurements prevents confirmation that high MB removal reflects true mineralization rather than surface accumulation. The ZnO-ZnTe hierarchical superstructures^48^ similarly exploit a multi-scale morphological architecture, with the increased surface roughness characteristic of superstructured materials providing a greater density of step-edge and kink-site active positions relative to smooth nanoparticles. Across the ZnO family, the convergence of pore size control and hierarchical or anisotropic morphology (whether nanorods, flower-like assemblies, or superstructures) consistently amplifies both the adsorption pre-concentration of MB and the spatial organization of charge carriers at the heterojunction interface.

### Bismuth-based systems

Bismuth-based photocatalysts represent a class where pore architecture data are conspicuously absent from most of the studies reviewed, and where the gap between morphological observation and textural quantification is most consequential. The co-precipitation-synthesized BiVO_4_ nanoparticles^126^ and the polyacrylamide gel-derived BiVO_4_^127^ both achieved high MB degradation efficiencies (up to 96.5%) under visible light, yet neither study provides BET surface area, pore volume, or pore size distribution data. The smooth, sphere-like morphology reported for gel-derived BiVO_4_ by SEM/TEM analysis implies a predominantly non-porous or low-porosity surface, which shifts the mechanistic emphasis entirely toward photocatalytic oxidation rather than adsorption-assisted degradation. Without pore-mediated pre-concentration of MB at the catalyst surface, the photocatalytic efficiency becomes more sensitive to light absorption characteristics and charge carrier dynamics. The omission of textural characterization in bismuth-based studies is a structural weakness in the field, as it prevents any assessment of whether porosity engineering could further enhance bismuth photocatalyst performance.

The morphological picture across the bismuth family is, however, more diverse than the textural data alone suggest. The gel-derived BiVO_4_ nanoparticles^105^ exhibit a smooth, sphere-like morphology by SEM analysis, implying a predominantly non-porous external surface with limited surface roughness. Without surface corrugation or porosity to pre-concentrate MB near active sites, the system relies entirely on photocatalytic oxidation at a geometrically smooth surface, reducing the effective collision frequency between MB and photogenerated ROS. The 96.5% degradation efficiency reported for this system therefore reflects the intrinsic photocatalytic activity of BiVO_4_ under the tested conditions rather than any morphological enhancement. The Bi_2_MoO_6_/LaFeO_3_ composite^55^ presents a different and more sophisticated approach, with a hierarchically organized morphology in which two-dimensional Bi_2_MoO_6_ nanosheets are immobilized on electrospun one-dimensional LaFeO_3_ hollow nanofibers. This 2D-on-1D architecture creates a high-density heterojunction interface where the large lateral area of the nanosheets contacts the curved nanofiber surface at multiple points, thereby generating spatially distributed junction sites rather than the localized contacts characteristic of particle-particle composites. The electrospun nanofiber backbone additionally provides a mechanically stable, high-porosity scaffold that resists the aggregation-driven surface area loss common in free-standing nanosheet systems. The interlayer-expanded MoS_2_/Bi_2_WO_6_ nanosheets^51^ use the high active site density inherent to 2D lamellar architectures, where the entire basal plane surface is theoretically accessible. However, the tendency of 2D materials toward restacking is acknowledged in neither study, and post-reaction SEM analysis confirming morphological integrity after multiple degradation cycles is absent. The absence of BET textural data across most bismuth systems, combined with the failure to monitor post-reaction morphological integrity, leaves a fundamental gap in the structure-performance assessment of this material family.

### g-C_3_N_4_-based systems

g-C_3_N_4_-based composites exhibit the broadest diversity of pore architectures and surface morphologies across the materials surveyed, comprising nanoscale porosity, macroscale 3D-printed structures, and the full morphological range from planar sheets to microtubular geometries. At the nanoscale, microtubular nanoporous g-C_3_N_4_@Ag (MN-g-C_3_N_4_@Ag)^128^ features a unique tubular morphology with nanoscale porosity that enables axial charge transport and high MB adsorption, with a Langmuir maximum adsorption capacity of 272.0 mg/g. The nanoporous tubular structure increases the effective surface area accessible to MB while also providing directional charge carrier pathways that spatially separate oxidation and reduction half-reactions. This architecture is mechanistically distinct from conventional planar g-C_3_N_4_ sheets and accounts, in part, for the 59-fold improvement in photocatalytic rate constant relative to bulk g-C_3_N_4_. The SnO_2_@g-C_3_N_4_ system^129^ similarly exploited the porous morphology of g-C_3_N_4_ sheets, with BET analysis confirming an enhanced surface area for the 5.0% SnO_2_@g-C_3_N_4_ composite compared to the individual components. The 97% MB degradation in 45 min was explicitly linked by the authors to both porous morphology and improved charge separation at the SnO_2_/g-C_3_N_4_ interface.

At the macroscale, the 3D-printed porous g-C_3_N_4_/geopolymer (C_3_N_4_/GP) composites^130^ demonstrate a qualitatively different strategy (i.e., the introduction of macropores through a K12 pore-forming agent during additive manufacturing). The resulting macroporous 3D architecture facilitates MB transport through the bulk material and increases the fraction of g-C_3_N_4_ active sites accessible under illumination, contributing to 94.62% MB removal within 60 min at 3 wt % g-C_3_N_4_ loading. While macropores (>50 nm) contribute negligibly to surface area compared to mesopores, their role in convective mass transport and in sustaining photocatalytic performance in flow-through configurations should not be underestimated. The morphological and textural diversity of g-C_3_N_4_-based systems is thus unique within this review. It is the only material family in which both the nanoscale pore geometry (here, axial channels in microtubular architectures) and the macroscale structural organization (here, 3D-printed geopolymer composites) are co-engineered for MB removal, rather than being incidental outcomes of synthesis conditions. This design intentionality is a distinguishing characteristic of the g-C_3_N_4_ family and warrants broader adoption across other material classes.

### MXene-integrated composites

MXene-based composites introduce a qualitatively different structural environment, arising from the inherent two-dimensional lamellar character of Ti_3_C_2_T_x_ sheets and the porosity introduced by incorporated functional components. The Ni-MOF/MXene (NM@MX) composite^131^ exhibits a BET surface area of 25.3 m^2^/g with a mesoporous structure and a pore size of approximately 31 nm, considerably larger than the TiO_2_-mesopore systems discussed above. This moderate surface area belies an exceptional MB adsorption capacity of 1012.37 mg/g, attributable to the synergistic contribution of electrostatic interactions, π–π stacking, and hydrogen bonding between MB and the composite’s functional groups. These mechanisms are enabled by the accessibility of internal adsorption sites within the mesopore network. The bimetallic MCuFe-MOF/PMMA@MXene (E-MPM) microspheres^132^ offer a further advance through a three-dimensional microsphere architecture assembled via electrostatic self-assembly, which enhances both pollutant adsorption capacity and mass transfer kinetics simultaneously. Achieving 91.9% MB degradation in 60 min under visible light, the E-MPM system illustrates that three-dimensional pore organization at the macroscopic scale can compensate for lower intrinsic surface area by improving reactant-catalyst contact frequency and ROS utilization efficiency.

From a morphological point of view, MXene-integrated composites introduce unique considerations arising from the two-dimensional lamellar character of Ti_3_C_2_T_x_. The E-MPM bimetallic microsphere architecture assembles MOF-derived components and MXene sheets into three-dimensional microspheres via electrostatic self-assembly. This creates a hierarchical architecture in which the macroscopic microsphere geometry facilitates catalyst recovery while the internal MXene/MOF interface provides the high-surface-area adsorption and charge-transfer environment responsible for the 91.9% MB degradation in 60 min. The 0D/1D/0D morphology of the CdS/ZnWO_4_/ZnS dual Z-scheme system^60^ is also significant in this context. The intimate point-contacts between 0D nanoparticles and 1D nanorods minimize interfacial resistance at each Z-scheme junction, thereby maximizing the rate of selective carrier recombination. A critical concern across all MXene-integrated and 2D-material-based composites is the tendency of lamellar sheets toward restacking under real operating conditions, which progressively reduces the effective contact area and impedes MB access to active interfacial sites. Post-reaction electron microscopy confirming lamellar integrity after multiple cycles is absent from the MXene literature reviewed here, and this gap represents a consequential unresolved question for any claim of practical durability.

### Hierarchy of morphological and textural design principles

Integrating the pore architecture and morphological data across all five material families reveals a set of structure-performance relationships that transcend individual material systems and constitute design principles for the field.

Narrow mesopores (∼3–14 nm) maximize surface area and promote adsorption pre-concentration but can introduce diffusion limitations for the bulky MB molecule. Larger mesopores (30–80 nm) reduce this diffusion barrier at the cost of surface area, improving MB accessibility to catalytically active sites. Macropores and 3D-printed architectures optimize mass transport in bulk configurations but contribute minimally to adsorption-driven removal. Nanoporous tubular g-C_3_N_4_ represents a distinct case where pore geometry enables directional charge transport in addition to enhanced adsorption. Morphologically, systems that simultaneously maximize heterojunction interfacial contact area, provide hierarchical porosity for MB pre-concentration, and support directional charge transport consistently deliver superior MB degradation performance, irrespective of the material family from which they are drawn. The chrysanthemum TiO_2_@In_2_S_3_, the 2D-on-1D Bi_2_MoO_6_/LaFeO_3_, the microtubular g-C_3_N_4_@Ag, and the 3D microsphere E-MPM system each realize a different expression of this principle through different synthetic strategies.

Two persistent limitations cut across all material classes. First, the inconsistent or absent reporting of pore isotherm type alongside pore diameter data prevents meaningful mechanistic differentiation between materials that share similar degradation efficiencies but may operate through fundamentally different surface interactions. Second, the persistent failure to report post-reaction SEM analysis confirming morphological stability is one of the field’s most consequential omissions. A photocatalyst that achieves excellent degradation in its first cycle but undergoes aggregation, restacking, or structural collapse upon reuse has no practical value, regardless of its initial performance metrics. Addressing both of these reporting gaps (i.e., standardized textural characterization and post-reaction morphological verification) must become a field-wide expectation before structure-performance claims in this literature can be considered complete.

## Mechanistic evaluation

The photocatalytic removal of MB is not a single-step phenomenon but an analytically connected chain. The identity and yield of ROS generated at the heterojunction interface determine which degradation pathways are activated, the cascade of intermediates produced along those pathways governs how much of the original organic carbon is ultimately mineralized, and the kinetic expression of all these processes is what performance benchmarking data in Table 1 reflect. Yet the four dimensions of this chain, i.e., ROS generation, degradation pathways, benchmarked performance, and kinetic constraints, have typically been reported in isolation across the MB photocatalysis literature. The sub-sections below present them as the integrated analytical sequence they are.

### ROS identity, generation mechanisms, and junction-type dependence

The identity and relative contribution of ROS in MB degradation are not uniform across heterostructured photocatalyst systems. They are determined by the thermodynamic properties of the component semiconductors, the junction type, and the redox potentials retained by photogenerated charge carriers after interfacial transfer. Understanding this variation is essential for rationally designing catalysts capable of achieving deep MB mineralization.

#### The principal ROS and their mechanistic roles

The photocatalytic degradation of MB proceeds through three ROS, including hydroxyl radicals (·OH), superoxide radical anions (·O_2_^−^), and photogenerated holes (h^+^), acting in concert through sequential oxidative reactions. The dominant and most mechanistically consequential of these is ·OH. DFT calculations have confirmed that ·OH-mediated demethylation of the dimethylamino groups of MB is thermodynamically highly favorable, with a reaction energy of −154 kcal/mol, establishing it as the kinetically dominant initial attack pathway.^133^ This stepwise demethylation generates the phenothiazine intermediates Azure B, Azure A, Azure C, and Thionine in succession, each retaining visible-light absorption capacity and, in the case of Azure B and Azure A, documented biological activity.^134^ The second major ·OH-initiated route involves oxidation of the central sulfur atom to form a sulfoxide intermediate, triggering ring opening of the tricyclic aromatic core and initiating fragmentation to low-molecular-weight carboxylic acids and inorganic ions.^134^^,^^135^ The ·O_2_^−^ radical, generated by one-electron reduction of dissolved O_2_ by CB electrons, plays a complementary reductive role. It can participate directly in ring-opening reactions and also serves as a precursor to H_2_O_2_, which upon further reduction yields additional ·OH. Photogenerated holes with sufficiently positive VB potentials can oxidize MB directly, thereby bypassing radical intermediaries, and this pathway becomes proportionally more significant when the VB position exceeds approximately +2.4 V vs. NHE.

#### Junction-type dependence of ROS generation

The composition of the ROS pool generated is shaped by heterojunction architecture, and this is where material-to-material differences are most consequential. In Type II heterojunctions, charge separation is achieved by migrating electrons to the lower CB and holes to the higher VB of the composite. This spatial separation comes at an energetic cost. The transferred electrons occupy a CB with reduced reduction potential, while the accumulated holes reside in a VB with diminished oxidation power. The practical consequence for MB degradation is illustrated by the WO_3_/TiO_2_ system, where the energetic penalty of Type II electron transfer directly constrains ·O_2_^−^ generation rates (Section TiO2-based heterostructures). The Bi_2_WO_6_/WO_3_ Type II system incurs the same penalty, requiring 6 h for 94.1% MB removal.^49^ Critically, Type II systems may generate ·OH primarily through direct hole oxidation of water rather than through the ·O_2_^−^ → H_2_O_2_ → ·OH route. This means that the mechanistic pathway is altered by junction type, with implications for which MB degradation intermediates accumulate and at what concentrations.

Z-scheme and S-scheme heterojunctions fundamentally change this by selectively eliminating the weaker carriers while preserving the strongest reductant and oxidant in each component. In Z-scheme systems such as CdS/ZnWO_4_/ZnS (a dual Z-scheme architecture achieving ∼100% MB removal in 50 min), the strongest reductant (CdS CB) and strongest oxidant (ZnS VB) are simultaneously retained, thereby enabling robust generation of both ·O_2_^−^ and ·OH in parallel.^60^ S-scheme systems operate through a built-in IEF that drives the same selective recombination with an explicit thermodynamic rationale. S-scheme photocatalysts including TiO_2_@In_2_S_3_,^40^ MnMgPO_4_/g-C_3_N_4_,^66^ and FeOOH QDs/CuFe_2_O_4_^81^ achieve superior radical yields compared to Type II counterparts on similar material platforms. EPR measurements, where reported, are expected to confirm the simultaneous generation of both ·OH (from the oxidation photocatalyst VB) and ·O_2_^−^ (from the reduction photocatalyst CB).

#### Material-specific deviations and anomalous ROS pathways

Beyond junction-type generalizations, several material-specific ROS profiles reported in this review diverge from the canonical ·OH/·O_2_^−^ picture. The ZnO/CuO p–n heterostructure generates ·O_2_^−^ from electrons accumulated in the ZnO CB alongside direct oxidation by h^+^ from the CuO VB, with scavenger results confirming that both pathways contribute meaningfully to MB removal.^44^ The g-C_3_N_4_(p)/AgCl system introduces Cl· radicals generated from photoproduced AgCl surface species, which supplements ·OH and ·O_2_^−^ and shifts the selectivity of the oxidative attack.^73^ The CuCo_2_S_4_/ZnO system combines photocatalysis with persulfate activation, making sulfate radicals (SO_4_·^-^; E^o^ = 2.5–3.1 V vs. NHE) the primary oxidizing species. Because both photocatalytically generated ROS and persulfate-derived SO_4_·^-^ may contribute to pollutant degradation, the study does not clearly distinguish the individual roles of these pathways, thereby limiting its value for assessing either mechanism independently.^43^ In contrast, the graphene-supported LFV composite (ESP/LFV) identifies ·O_2_^−^ as the dominant oxidizing species through scavenger experiments, while ·OH and h^+^ contribute secondarily. This behavior is attributed to graphene’s electron-trapping capability, which suppresses charge recombination and promotes O_2_ reduction over direct hole-driven oxidation.^118^

As established in Section characterization standards for mechanistic assignment, scavenger data alone cannot resolve charge transfer direction or the spatial origin of radicals within the heterojunction, and EPR spin-trapping and operando XPS remain necessary for definitive ROS characterization.

### MB degradation pathways, intermediate products, and mineralization extent

A critical but frequently overlooked dimension of photocatalytic MB removal studies is the question of what actually happens to the MB molecule during and after the reaction. Most of the papers in the heterostructured photocatalysis literature report removal efficiency based exclusively on the attenuation of absorbance at 664 nm. While this metric is convenient and widely adopted, it measures decolorization, not degradation, and certainly not mineralization. Decolorization occurs when the chromophoric functional groups of the MB molecule are disrupted, abolishing visible color, but substantial quantities of colorless organic fragments and potentially toxic intermediates can persist in solution well after the 664 nm signal has vanished. This distinction has profound implications for environmental safety and for the honest evaluation of photocatalytic performance.

MB is a phenothiazine-based cationic dye with a complex aromatic core that includes a central heterocyclic ring system flanked by two dimethylamino-substituted benzene rings bridged by a sulfur atom. Its degradation by ROS, predominantly ·OH, ·O_2_^−^, and h^+^, does not proceed in a single step but through a cascade of sequential oxidative transformations that progressively reduce the molecular complexity of the parent compound. Understanding this cascade is essential for assessing whether a given photocatalytic system achieves true environmental remediation.

#### Primary attack pathways

**Figure 9:**
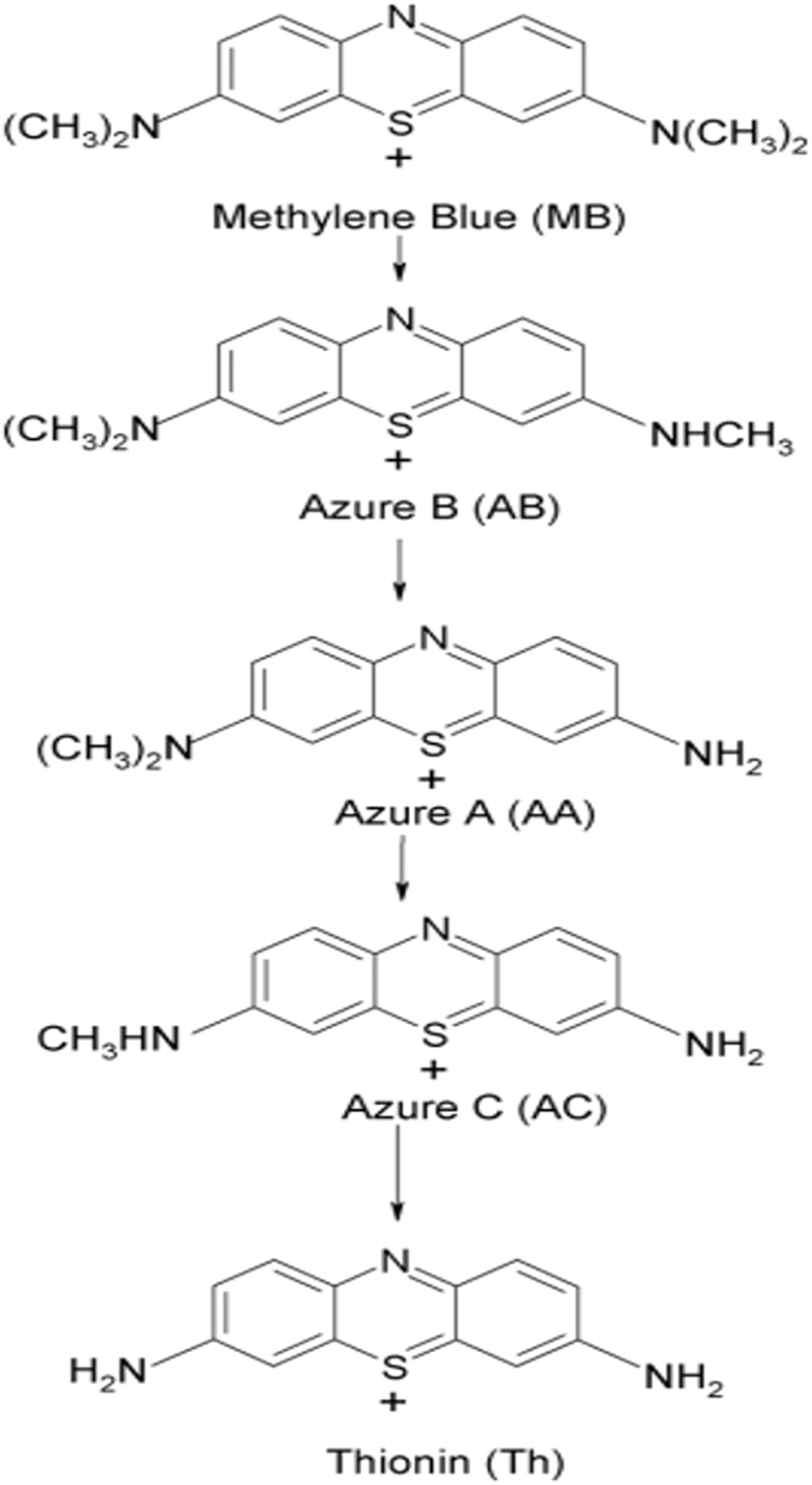
Schematic representation of the demethylation pathway of methylene blue during photocatalytic degradation (reproduced from Wang et al.^41^ Copyright 2010 Elsevier)

The photocatalytic degradation of MB has been established to proceed through two principal initial attack pathways, both elucidated by GC/MS and LC–UV/Vis–MS analysis. The first and most energetically favored pathway is demethylation, in which hydroxyl radicals sequentially remove the N-methyl groups from the dimethylamino substituents of the MB molecule.^134^^,^^135^ DFT calculations have confirmed that demethylation mediated by ·OH is thermodynamically highly favorable, with a reaction energy of −154 kcal/mol, supporting its role as the kinetically dominant initial step.^133^ This stepwise demethylation generates a series of structurally related phenothiazine intermediates with progressively decreasing molecular masses (Figure 9), including Azure B (trimethyl thionine, m/z = 270), Azure A (asymmetric dimethyl thionine, m/z = 256), Azure C (asymmetric monomethyl thionine, m/z = 242), and Thionine (m/z = 228).^4^ Each of these compounds retains the intact phenothiazine core and visible light absorption capacity, meaning that they continue to absorb in the visible region even as MB itself is consumed. Crucially, Azure B and Azure A are biologically active compounds documented to interact with cellular membranes and nucleic acids, raising ecotoxicological concerns if they persist in treated effluents. The second major attack pathway involves sulfoxide formation, in which the central sulfur atom of the phenothiazine ring system is oxidized to yield a sulfoxide intermediate, identified by GC/MS, which serves as a gateway to central ring opening and initiates breakdown of the tricyclic aromatic core.^134^

#### Intermediate products and toxicological implications

Following demethylation and initial ring attack, subsequent oxidative steps lead to rupture of the central heterocyclic ring, generating a series of smaller aromatic and aliphatic intermediates. These include hydroxylated benzene derivatives, phenolic compounds, naphthoquinone-type structures, and various substituted aromatic amines.^134^^,^^135^ The aromatic amines are of particular toxicological concern. Compounds such as 4-aminophenol and structurally related primary aromatic amines are classified as mutagenic and carcinogenic, and their transient accumulation in photocatalytic reaction mixtures represents a period during which treated water may actually be more acutely hazardous than the original MB solution.^136^ This intermediate toxicity peak has received almost no attention in the heterostructured photocatalysis literature reviewed here, despite its critical practical importance.

Continued ·OH attack on the hydroxylated aromatic intermediates drives successive ring hydroxylation and ultimately ring-opening reactions, converting the aromatic fragments into low-molecular-weight aliphatic compounds. These include short-chain carboxylic acids (oxalic acid, acetic acid, formic acid, maleic acid), aldehydes (formaldehyde, acetaldehyde), and inorganic ions. The heteroatoms of MB (nitrogen, sulfur, and chlorine) undergo parallel oxidative transformations. Nitrogen atoms in the −3 oxidation state, present in amino groups, are converted first to NH_4_^+^ and subsequently, more slowly, to NO_3_^−^. The central sulfur bridge is oxidized progressively through sulfoxide and sulfone intermediates to eventually yield SO_4_^2−^, while Cl^−^ is released as the inorganic counterion. The terminal products of complete mineralization are therefore CO_2_, H_2_O, NH_4_^+^/NO_3_^−^, SO_4_^2−^, and Cl^−^. Acetic acid is notably recalcitrant and represents the rate-limiting step in full mineralization. Its conversion to CO_2_ proceeds through repeated photo-Kolbe-type decarboxylation reactions and is typically the slowest stage of the overall degradation sequence.^134^

#### The decolorization-mineralization gap and the dye-sensitization complication

The distinction between dye decolorization, degradation, and complete mineralization is best evaluated using TOC and COD measurements. However, these parameters are rarely reported in studies of heterostructured photocatalysts. Among the few studies that include them, TOC removal is substantially lower than decolorization efficiency, highlighting the persistence of organic intermediates after the disappearance of color.

In the Mg–Al LDH@g-C_3_N_4_@Ag_3_PO_4_ ternary heterostructure reported by Salehi et al., scavenger experiments using isopropanol (IPA), EDTA-2Na, and benzoquinone (BQ) identified ·OH, h^+^, and ·O_2_^−^ as the principal reactive species involved in MB degradation, with ·OH acting as the dominant oxidant. The CB and VB edge potentials of the individual semiconductors were estimated using the Mulliken electronegativity method according to Equations 1 and 2.

The absolute electronegativity (*X*) was determined from the arithmetic mean of the atomic ionization energy (*E*_ion_) and electron affinity (*E*_EA_) of the constituent elements, as expressed in Equation 3:

(Equation 3) $$X=0.5\left(E_{\text{EA}}+E_{\text{ion}}\right)$$

**Figure 10:**
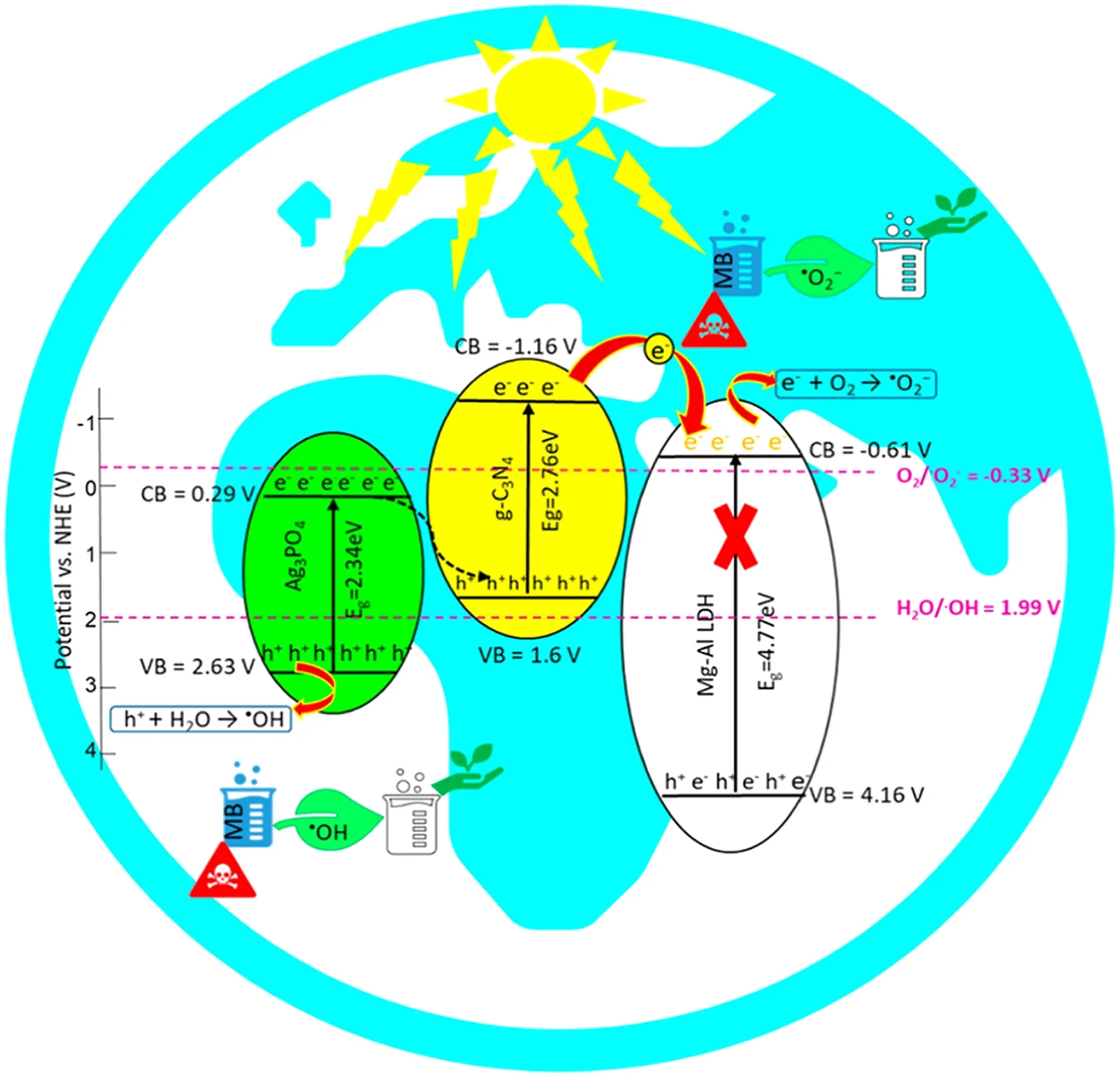
Proposed photocatalytic degradation mechanism of MB by Mg–Al LDH@g-C_3_N_4_@Ag_3_PO_4_, showing CB/VB alignment, selective carrier recombination, and ROS generation pathways (reproduced from Salehi et al.^36^ under the CC-BY-NC-ND 4.0 license. Copyright 2024 American Chemical Society)

Using Equations 1, 2, and 3, the VB potential of Ag_3_PO_4_ was calculated to be +2.63 V versus NHE, exceeding the thermodynamic potential required for ·OH generation through water oxidation (+1.99 V versus NHE). This confirms that direct hole-driven ·OH formation is thermodynamically favorable. As illustrated in Figure 10, the proposed mechanism involves the recombination of CB electrons from Ag_3_PO_4_ with VB holes from g-C_3_N_4_, thereby preserving the highly oxidizing VB holes of Ag_3_PO_4_ and the strongly reducing CB electrons of g-C_3_N_4_. Under visible-light irradiation, both semiconductors generate electron-hole pairs. The CB electrons of Ag_3_PO_4_ recombine with the VB holes of g-C_3_N_4_, leaving the VB holes of Ag_3_PO_4_ (+2.63 V vs. NHE) to produce ·OH radicals and the CB electrons of g-C_3_N_4_ to generate ·O_2_^−^ radicals. Despite achieving 99% MB removal, TOC analysis showed that only 88% of the initial organic carbon was mineralized.^36^ The remaining 12% persisted as colorless organic intermediates that were undetected by the 664 nm absorbance measurement used to determine dye removal. This result clearly demonstrates that complete decolorization does not necessarily indicate complete mineralization.

A similar trend was observed for a CeO_2_ nanoparticle/graphene oxide/polyacrylamide composite, where approximately 90% MB decolorization under UV-A irradiation was accompanied by lower TOC and COD removal efficiencies, indicating the persistence of colorless degradation products after the disappearance of the visible absorption peak.^35^ These findings demonstrate that UV–Vis absorbance measurements consistently overestimate the extent of mineralization and may therefore provide an environmentally misleading assessment of photocatalytic performance.

Dye-sensitized degradation further complicates performance interpretation under visible light, as apparent activity toward MB may partly reflect dye self-sensitization rather than intrinsic semiconductor photoactivation.

### Performance benchmarking and comparative kinetic analysis

Degradation efficiency depends on initial MB concentration, catalyst loading, solution volume, light source power and spectrum, and irradiation geometry. A system achieving 99% removal of 5 mg/L MB under a 300 W xenon lamp is not directly comparable to one achieving 95% removal of 20 mg/L MB under a 30 W LED, yet both entries appear in Table 1 and are routinely cited as evidence of high performance. Normalizing performance by pseudo-first-order rate constant (k, min^−1^) partially addresses this issue (and the k values in Table 1 show considerably more spread than the raw efficiency data), but even rate constants depend on catalyst loading and light intensity, and are rarely reported per unit mass of catalyst or per unit incident photon flux. Apparent quantum yields, which would permit true photonic efficiency comparison, are reported in almost none of the studies reviewed here, and this represents a significant gap in reporting standards across the field.

With these in mind, the dataset in Table 1 supports the following conclusions:

i. Z-scheme and S-scheme configurations consistently achieve the highest combination of degradation rate and efficiency, with many systems recording >95% MB removal within 30–60 min.
ii. Type II systems show broader performance variance, with some achieving comparable efficiency but generally requiring longer irradiation times or higher catalyst loadings.
iii. p–n heterojunctions occupy an intermediate performance tier, with the advantage of well-understood and chemically verifiable junction physics.
iv. Ternary systems cluster toward the upper performance range, though this advantage is not always clearly separable from the larger surface areas and greater light absorption cross-sections of more complex morphologies.

### Kinetic limitations and the sub-20-min barrier

One of the most consequential yet underacknowledged limitations of the heterostructured photocatalyst literature surveyed in this review is the near-universal failure to achieve substantial MB removal within 20 min of irradiation. Table 1 reveals that, across the binary and ternary heterostructured systems spanning TiO_2_-, ZnO-, bismuth-based, g-C_3_N_4_-, and MXene-integrated platforms, the minimum irradiation time required for >90% MB removal is 30 min at best. The median across the dataset is substantially higher, typically falling in the 60–120 min range. This kinetic gap between laboratory performance and the operational demands of realistic wastewater treatment systems represents a fundamental but rarely foregrounded design challenge that demands critical attention.

The 30-min threshold is itself achieved only by the highest-performing systems. Among the studies reviewed, CeO_2_@PDA/BiOBr is the most rapid Z-scheme system reported, achieving 100% MB removal (30 mg/L) in 30 min under 300 W xenon lamp irradiation, with a rate constant of 0.1017 min^−1^.^93^ The Co-NiS/S-g-C_3_N_4_ 1D/2D Z-scheme heterostructure achieves approximately 98% MB removal in 32 min.^95^ These represent the upper performance boundary across the entire reviewed dataset. Most of the systems, including mechanistically sophisticated architectures such as MnMgPO_4_/g-C_3_N_4_ (S-scheme, 40 min),^66^ CdS/ZnWO_4_/ZnS (dual Z-scheme, 50 min),^60^ and Fe_3_O_4_/TiO_2_/g-C_3_N_4_ (Z-scheme, 80 min),^62^ fall well outside the sub-20-min window. Type II systems are generally the slowest, with exemplars such as WO_3_/TiO_2_ (87.8% in 150 min),^41^ CuO/ZnO (98.5% in 150 min),^44^ and MoS_2_/CdS/Bi_2_MoO_6_ (96% in 120 min)^61^ requiring irradiation periods that would be operationally prohibitive in continuous-flow treatment scenarios. The Bi_2_WO_6_/WO_3_ Type II system is especially striking in this respect, requiring a full 6 h for 94.1% MB removal.^49^ This timeline is incompatible with any realistic treatment application.

The kinetic limitations observed in MB photodegradation are fundamentally mechanistic rather than incidental and arise from three interrelated factors. First, an induction period associated with adsorption equilibration is commonly required. Most studies reviewed herein employ a dark adsorption stage of 30–60 min before irradiation to allow MB molecules to accumulate on the photocatalyst surface, thereby enhancing subsequent photodegradation efficiency. This process is further hindered by pore diffusion limitations, particularly in mesoporous materials with pore diameters of 3–14 nm. Given the relatively large molecular size of MB (∼1.43 nm), diffusion through these pores is restricted, delaying the establishment of the surface concentration gradient necessary for rapid degradation.

Second, degradation rates are constrained by the intrinsic rate of ROS generation under the applied irradiation conditions. Most studies summarized in Table 1 use high-intensity xenon lamps (300–500 W), which provide photon fluxes substantially greater than those available under natural sunlight or low-power LED illumination. Nevertheless, even under these intensified conditions, complete degradation is rarely achieved within 20 min. Systems evaluated under solar irradiation, such as ZnO–ZnTe superstructures, achieved approximately 91% MB removal after 120 min,^48^ indicating generally slower kinetics and highlighting the limited transferability of laboratory-derived rate constants to practical solar-driven applications.

Third, the energetics of charge-transfer junctions impose additional constraints. As discussed earlier, Type II heterojunctions reduce the redox potentials of transferred charge carriers, thereby suppressing ·O_2_^−^ and ·OH generation and slowing degradation. Although Z-scheme and S-scheme heterojunctions retain stronger redox potentials, efficient MB removal still requires sufficient time for sequential oxidation processes (including demethylation, ring opening, carboxylic acid formation, and eventual mineralization) to reach completion.

The sole system in this review that approaches sub-20-min performance is the ESP/LFV composite (graphene-coated cotton fabric supporting LaFe_0.94_V_0.01_Mn_0.05_O_3_ nanoparticles), which achieved 96.3% MB degradation in 10 min under direct solar irradiation.^118^ However, this study was demonstrated at a single MB concentration (20 ppm) in tap water, recyclability data for the fabric composite were not reported, and the graphene substrate’s long-term structural integrity under repeated solar exposure was not systematically evaluated. These omissions substantially limit the generalizability of this result.

The convergent implication is that the field has optimized primarily for maximum degradation efficiency at the expense of kinetic rate, and has done so under idealized single-pollutant, low-matrix, high-photon-flux conditions that do not reflect deployment constraints. Genuinely achieving sub-20-min MB removal at practically relevant catalyst loadings, MB concentrations, and under solar irradiation will require a shift toward co-engineering of heterojunction architecture with mass transport design (hierarchical pore structures, flow-through reactors), plasmonic enhancement for instantaneous photon harvesting, and pre-adsorptive concentration mechanisms that eliminate the dark-period kinetic delay. Until these design dimensions are integrated systematically, the sub-20-min barrier will remain a defining limitation of this technology class.

## Scalability, cost, and future directions

The performance data compiled across this review demonstrate that heterostructured photocatalysts consistently achieve MB degradation efficiencies exceeding 90% under visible light, with the most advanced Z-scheme and S-scheme architectures approaching complete removal within 30–60 min. However, the translation of these laboratory achievements into deployable water treatment technologies is constrained by a set of cost, scalability, and operational barriers that the field has been slow to address systematically. A critical appraisal of the materials and synthesis routes reviewed here reveals that economic and manufacturing considerations are almost entirely absent from individual study design. No study in this review reports a techno-economic analysis, material cost estimate, or life cycle assessment alongside photocatalytic performance data. This silence allows researchers to claim practical relevance on the basis of degradation efficiency alone, without confronting the economic realities that determine whether a technology can leave the laboratory.

### Precursor cost and critical material dependence

The single most pervasive cost barrier across the reviewed systems is the use of noble metals and rare elements as functional components. Silver is incorporated as a plasmonic enhancer, electron mediator, or active phase in multiple high-performing systems, including Ag/g-C_3_N_4_/LaFeO_3_,^87^ Ag@ZnO@Ti_3_C_2_,^99^ Ag_3_PO_4_/N–TiO_2_ nanotubes,^37^ and g-C_3_N_4_/Ti_3_C_2_T_x_/Ag_3_PO_4_.^71^ Silver photocatalysts are susceptible to photoreduction to Ag^0^ under illumination. This degradation pathway simultaneously reduces catalytic activity and creates secondary contamination. However, none of these studies quantify the economic cost of silver incorporation or assess the feasibility of silver recovery and recycling at scale. The BiVO_4_:Er^3+^,Yb^3+^/BiOCl full-spectrum S-scheme system^105^ compounds this issue by incorporating rare earth elements (erbium and ytterbium) whose global supply chains are geographically concentrated and strategically restricted, rendering large-scale deployment contingent on geopolitical factors entirely outside the photocatalyst designer’s control.

Cadmium-containing systems represent a distinct but equally serious barrier, grounded in regulatory rather than cost constraints. Multiple high-performing systems in this review incorporate CdS as the narrow-bandgap visible-light absorber. These include CdS/ZnWO_4_/ZnS,^60^ MoS_2_/CdS/Bi_2_MoO_6_,^61^ g-C_3_N_4_/CdS,^88^ TiO_2_-CdS/bacterial cellulose,^38^ Bi/CdS/TiO_2_,^103^ and Co_x_Ni_1-x_TiO_3_/CdS.^96^ Cadmium is a classified carcinogen and the subject of increasingly stringent international regulation under frameworks including the EU RoHS Directive and the Stockholm Convention. The bacterial cellulose matrix in the TiO_2_-CdS system was noted to physically sequester leached Cd^2+^ ions, which addresses catalyst stability but does not resolve the main regulatory problem (i.e., a photocatalyst that generates cadmium-contaminated sludge as a byproduct of wastewater treatment exchanges one pollutant for another).

### Synthesis route scalability

Beyond precursor cost, the synthesis routes used across this literature are almost uniformly unsuitable for mass production without fundamental re-engineering. Hydrothermal and solvothermal methods dominate, appearing in the preparation of WO_3_/TiO_2_ (Wang et al., 2021), Bi_2_WO_6_/TiS_2_,^50^ ZnO/CuO,^44^ BiOBr/ZnO/BiOI,^59^ and numerous others. These routes require sealed autoclave reactors, precise temperature and pressure control, and prolonged reaction times, which translate into high capital investment and limited batch throughput at industrial scale. The synthesis of MXene-integrated composites (Ag@ZnO@Ti_3_C_2_, g-C_3_N_4_/Ti_3_C_2_T_x_/Ag_3_PO_4_) introduces another form of complexity. Ti_3_C_2_T_x_ MXene itself is produced by selective HF etching of Ti_3_AlC_2_ MAX phase. This process is hazardous, difficult to scale reproducibly, and generates toxic fluoride waste streams. Neither study addresses these upstream manufacturing constraints.

### Lower-cost alternatives and their limitations

A portion of the reviewed systems points toward more economically viable design strategies, though each comes with unresolved challenges. g-C_3_N_4_-based systems are among the most cost-accessible. g-C_3_N_4_ is readily synthesized by thermal condensation of urea or melamine (earth-abundant, inexpensive precursors) and performs as the backbone of several high-efficiency systems including Fe_3_O_4_/TiO_2_/g-C_3_N_4_,^62^ β-Bi_2_O_3_/g-C_3_N_4_,^56^ and MnMgPO_4_/g-C_3_N_4_.^66^ Similarly, biochar-integrated composites such as Mo–Bi_2_WO_6_/WO_3_/Biochar^68^ and g-C_3_N_4_/ferrite/biochar^86^ exploit agricultural waste streams, reducing raw material costs substantially. The waste-battery-derived Mn_0.6_Zn_0.4_Fe_2_O_4_@Zn_0.9_Mn_0.1_O photocatalyst^64^ and DBAA graphite recovered from spent alkaline batteries^119^ represent a circular economy approach with genuine cost merit. However, the photocatalytic activity of the DBAA system was partly attributed to residual Zn and Mn oxide impurities. This raises the same leaching concern that makes CdS-based systems problematic. It also illustrates that cost savings achieved through waste feedstocks may generate unquantified secondary environmental liabilities.

### Operational energy cost and irradiation infrastructure

A dimension of cost that is entirely unaddressed in this literature is the operational energy expenditure associated with the light sources used to drive photocatalysis. Most of the systems reviewed rely on 300–500 W xenon lamps. This energy intensity is incompatible with cost-effective wastewater treatment at scale. Only a small fraction of studies use solar irradiation,^48^^,^^64^ or low-wattage LEDs,^51^^,^^68^ and none quantifies the electrical energy per order of magnitude (EE/O). Without this metric, performance comparisons across the dataset are economically meaningless, and the claim that photocatalysis offers a more cost-effective alternative to coagulation-flocculation or adsorption cannot be substantiated. Reusability data across the dataset extend only to 3–5 cycles, whereas industrial photocatalytic systems must sustain stable operation for hundreds of cycles or continuous service over months to years. These gaps mean that the economic case for heterostructured photocatalysts in MB removal remains entirely unbuilt, and building it must become a research priority commensurate with mechanistic investigation.

### Conclusions, critical gaps, and future research priorities

This review has examined the design principles, mechanistic foundations, and photocatalytic performance of heterostructured systems for MB removal, spanning Type II, Z-scheme, S-scheme, p–n, and Schottky junction architectures across TiO_2_-, ZnO-, bismuth-based, g-C_3_N_4_-, and MXene-integrated material families. The evidence surveyed establishes beyond reasonable doubt that heterojunction engineering represents a decisive advance over single-component semiconductors. Binary and ternary composites consistently achieve MB degradation efficiencies exceeding 90% under visible light, with the highest-performing systems, CdS/ZnWO_4_/ZnS^60^ and CeO_2_@PDA/BiOBr,^93^ reaching near-complete removal within 30–50 min. S-scheme and Z-scheme architectures have emerged as the mechanistically superior configurations, preserving high-energy electrons and holes for simultaneous ·OH and ·O_2_^−^ generation while avoiding the energetic penalty inherent to conventional Type II systems. The S-scheme framework, supported by a well-defined IEF and verifiable band bending, offers the most theoretically coherent design logic for next-generation MB photocatalysts and is expected to increasingly displace the Type II approach as the field matures.

However, this review also exposes a set of structural deficiencies that prevent the field from realizing the practical potential implied by its performance data. Five critical gaps warrant explicit identification. The first and most pervasive is the inadequacy of mechanistic evidence. Across the literature reviewed, junction-type assignments (whether Type II, Z-scheme, or S-scheme) rest almost universally on indirect characterization. Mott–Schottky analysis, PL quenching, photocurrent measurements, and radical scavenger experiments. None of these methods, individually or in combination, can resolve the direction of charge flow across a heterojunction interface. Direct evidence from *in situ* XPS under illumination, EPR spin-trapping, femtosecond transient absorption spectroscopy, or KPFM is reported in only a minority of studies. Mechanistic claims that have not been subjected to this evidential standard should be understood as working hypotheses rather than established facts, and the adoption of these techniques as routine characterization components must become a field-wide priority.

The second gap concerns the conflation of decolorization with degradation. As demonstrated in Section the decolorization-mineralization gap and the dye-sensitization complication, the performance data compiled in Table 1 are based almost entirely on attenuation of absorbance at 664 nm, which is not sensitive to the colorless, potentially toxic intermediates that persist in solution after visible color disappears. The analytical requirements for closing this gap (i.e., TOC/COD kinetic profiling, LC-MS intermediate identification, and biotoxicity assays) are detailed in Section the decolorization-mineralization gap and the dye-sensitization complication and must become standard reporting components across the field.

The third gap is the kinetic shortfall identified in Section kinetic limitations and the sub-20-minute barrier. With the sole exception of the ESP/LFV graphene-fabric composite (96.3% in 10 min under solar irradiation,^118^ no system in the dataset achieves >90% MB removal in under 20 min. Closing this gap will require the deliberate co-engineering of heterojunction architecture with mass transport design (hierarchical pore structures, flow-through reactor geometries) alongside plasmonic enhancement for accelerated photon harvesting and adsorptive pre-concentration strategies that eliminate the 30–60 min dark equilibration period that currently precedes irradiation in most experimental protocols.

The fourth gap is the absence of an economic and scalability case. The widespread use of noble metals, rare earth dopants, cadmium-containing semiconductors, and MXene substrates synthesized by HF etching imposes cost, regulatory, and environmental barriers that the literature does not confront. No study reviewed here reports a techno-economic analysis, material cost estimate, EE/O metric, or life cycle assessment. Without these data, claims of practical relevance are unsubstantiated. Earth-abundant, cadmium-free, noble-metal-free systems represent the most credible design direction for economically viable deployment,^62^^,^^64^^,^^68^ but their long-term operational stability under realistic, multi-pollutant wastewater conditions has not been established beyond 3–5 reuse cycles.

The fifth gap is the predominant reliance on MB as a model pollutant under simplified laboratory conditions, typically involving single-component systems, neutral pH, and high photon flux. Consequently, the actual performance of these materials in real textile and industrial wastewater (characterized by turbidity, variable pH, competing contaminants, and ambient solar irradiation) remains largely unexplored.

Looking ahead, the priority research directions for the next 3–5 years include:

i. Standardization of mechanistic characterization protocols
ii. Mandatory inclusion of TOC/COD and intermediate analysis in performance reporting
iii. Reactor engineering for solar-driven, continuous-flow operation
iv. Techno-economic evaluation as an integral component of catalyst design, and
v. Systematic real-matrix testing under industrial conditions.

The integration of artificial intelligence and machine learning in photocatalyst screening, illustrated by the Ag/ZrO_2_/TCN system developed with ML-assisted optimization,^98^ offers a promising route to accelerating the discovery of high-performance, earth-abundant heterojunction combinations without exhaustive empirical trials. Until these directions are pursued with the same rigor currently applied to mechanistic photocatalysis, the gap between laboratory performance and deployable water treatment technology will remain the defining unfulfilled promise of this field.

## Acknowledgments

The authors are grateful to the 10.13039/501100001322South African Medical Research Council for the Self-Initiated Research (SIR) grant and the 10.13039/501100001343University of Pretoria Research Development Program for the financial support.

## Author contributions

D.C.A.: writing – review and editing, writing – original draft, methodology, data curation, and conceptualization. A.T.O.: writing – review and editing, writing – original draft, methodology, data curation, and conceptualization. B.O.O.: writing – review and editing and supervision. M.I.O.: writing – review and editing; T.L.Y.: writing – review and editing, project administration, investigation, data curation, and conceptualization.

## Declaration of interests

The authors declare no competing interests.

## References

[1] Wainwright M. (2008). Dyes in the development of drugs and pharmaceuticals. Dyes Pigm..

[2] Oz M., Lorke D.E., Hasan M., Petroianu G.A. (2011). Cellular and molecular actions of Methylene Blue in the nervous system. Med. Res. Rev..

[3] Brooks M.M. (1933). Methylene blue as antidote for cyanide and carbon monoxide poisoning. JAMA.

[4] Khan I., Saeed K., Zekker I., Zhang B., Hendi A.H., Ahmad A., Ahmad S., Zada N., Ahmad H., Shah L.A. (2022). Review on Methylene Blue: Its Properties, Uses, Toxicity and Photodegradation. Water.

[5] Oladoye P.O., Ajiboye T.O., Omotola E.O., Oyewola O.J. (2022). Methylene blue dye: Toxicity and potential elimination technology from wastewater. Results Eng..

[6] Hayat M., Shah A., Nisar J., Shah I., Haleem A., Ashiq M.N. (2022). A Novel Electrochemical Sensing Platform for the Sensitive Detection and Degradation Monitoring of Methylene Blue. Catalysts.

[7] Cusioli L.F., Quesada H.B., Baptista A.T.A., Gomes R.G., Bergamasco R. (2020). Soybean hulls as a low-cost biosorbent for removal of methylene blue contaminant. Environ. Prog. Sustain. Energy.

[8] Oussadi K., Al-Farraj S., Benabdallah B., Benettayeb A., Haddou B., Sillanpaa M. (2025). Wool keratin as a novel, alternative, low-cost adsorbent rich in various –N and –S proteins for eliminating methylene blue from water. Biomass Convers. Biorefin..

[9] Bhatia D., Sharma N.R., Singh J., Kanwar R.S. (2017). Biological methods for textile dye removal from wastewater: A review. Crit. Rev. Environ. Sci. Technol..

[10] Ihaddaden S., Aberkane D., Boukerroui A., Robert D. (2022). Removal of methylene blue (basic dye) by coagulation-flocculation with biomaterials (bentonite and Opuntia ficus indica). J. Water Process Eng..

[11] Tarpani R.R.Z., Azapagic A. (2018). Life cycle costs of advanced treatment techniques for wastewater reuse and resource recovery from sewage sludge. J. Clean. Prod..

[12] Koe W.S., Lee J.W., Chong W.C., Pang Y.L., Sim L.C. (2020). An overview of photocatalytic degradation: photocatalysts, mechanisms, and development of photocatalytic membrane. Environ. Sci. Pollut. Res..

[13] Orimolade B.O., Idris A.O., Feleni U., Mamba B. (2021). Recent advances in degradation of pharmaceuticals using Bi 2 WO 6 mediated photocatalysis – A comprehensive review. Environ. Pollut..

[14] Orimolade B.O., Arotiba O.A. (2020). Bismuth vanadate in photoelectrocatalytic water treatment systems for the degradation of organics: A review on recent trends. J. Electroanal. Chem..

[15] Low J., Yu J., Jaroniec M., Wageh S., Al-Ghamdi A.A. (2017). Heterojunction Photocatalysts. Adv. Mater..

[16] Wang H., Zhang L., Chen Z., Hu J., Li S., Wang Z., Liu J., Wang X. (2014). Semiconductor heterojunction photocatalysts: design, construction, and photocatalytic performances. Chem. Soc. Rev..

[17] Nazir A., Huo P., Wang H., Weiqiang Z., Wan Y. (2023). A review on plasmonic-based heterojunction photocatalysts for degradation of organic pollutants in wastewater. J. Mater. Sci..

[18] Okab A.A., Jabbar Z.H., Graimed B.H., Alwared A.I., Ammar S.H., Hussein M.A. (2023). A comprehensive review highlights the photocatalytic heterojunctions and their superiority in the photo-destruction of organic pollutants in industrial wastewater. Inorg. Chem. Commun..

[19] Jabbar Z.H., Graimed B.H. (2022). Recent developments in industrial organic degradation via semiconductor heterojunctions and the parameters affecting the photocatalytic process: A review study. J. Water Process Eng..

[20] Lai Y.-J., Lee D.-J. (2021). Pollutant degradation with mediator Z-scheme heterojunction photocatalyst in water: A review. Chemosphere.

[21] Dabhane H., Chatur S., Jadhav G., Tambade P., Medhane V. (2021). Phytogenic synthesis of gold nanoparticles and applications for removal of methylene blue dye: A review. Environ. Chem. Ecotoxicol..

[22] Mashkoor F., Nasar A. (2020). Magsorbents: Potential candidates in wastewater treatment technology – A review on the removal of methylene blue dye. J. Magn. Magn Mater..

[23] Santoso E., Ediati R., Kusumawati Y., Bahruji H., Sulistiono D.O., Prasetyoko D. (2020). Review on recent advances of carbon based adsorbent for methylene blue removal from waste water, Mater. Today Chem..

[24] Hamad H.N., Idrus S. (2022). Recent Developments in the Application of Bio-Waste-Derived Adsorbents for the Removal of Methylene Blue from Wastewater: A Review. Polymers.

[25] Yang Y., Zhu Q., Peng X., Sun J., Li C., Zhang X., Zhang H., Chen J., Zhou X., Zeng H., Zhang Y. (2022). Hydrogels for the removal of the methylene blue dye from wastewater: a review. Environ. Chem. Lett..

[26] Al-Nasiri K., Al-Rashidi S. (2026). Black phosphorus-based Heterostructures for visible-light-driven photocatalytic degradation of pollutants. Inorg. Chem. Commun..

[27] Orimolade B.O., Peleyeju M.G., Yusuf T.L. (2026). A comprehensive review on bismuth-based ternary heterojunctions in photocatalytic wastewater treatment. J. Environ. Manage..

[28] ONG S., TOORISAKA E., HIRATA M., HANO T. (2005). Biodegradation of redox dye Methylene Blue by up-flow anaerobic sludge blanket reactor. J. Hazard. Mater..

[29] Cruz-Rizo A., Gutiérrez-Granados S., Salazar R., Peralta-Hernández J.M. (2017). Application of electro-Fenton/BDD process for treating tannery wastewaters with industrial dyes. Sep. Purif. Technol..

[30] Sun Q., Saratale R.G., Saratale G.D., Kim D.-S. (2018). Pristine and modified radix Angelicae dahuricae (Baizhi) residue for the adsorption of methylene blue from aqueous solution: A comparative study. J. Mol. Liq..

[31] Li W., Chen S., Tian M., Ke G. (2024). Removal of methylene blue from dye wastewater by cattail leaf-based porous carbon materials. Environ. Tech. Rev..

[32] Lum P.T., Foo K.Y., Zakaria N.A., Palaniandy P. (2020). Ash based nanocomposites for photocatalytic degradation of textile dye pollutants: A review. Mater. Chem. Phys..

[33] Lebron Y.A.R., Moreira V.R., de Souza Santos L.V. (2021). Biosorption of methylene blue and eriochrome black T onto the brown macroalgae Fucus vesiculosus : equilibrium, kinetics, thermodynamics and optimization. Environ. Technol..

[34] Cwalinski T., Polom W., Marano L., Roviello G., D’Angelo A., Cwalina N., Matuszewski M., Roviello F., Jaskiewicz J., Polom K. (2020). Methylene Blue—Current Knowledge, Fluorescent Properties, and Its Future Use. J. Clin. Med..

[35] Kalaycıoğlu Z., Özuğur Uysal B., Pekcan Ö., Erim F.B. (2023). Efficient Photocatalytic Degradation of Methylene Blue Dye from Aqueous Solution with Cerium Oxide Nanoparticles and Graphene Oxide-Doped Polyacrylamide. ACS Omega.

[36] Salehi G., Bagherzadeh M., Abazari R., Hajilo M., Taherinia D. (2024). Visible Light-Driven Photocatalytic Degradation of Methylene Blue Dye Using a Highly Efficient Mg–Al LDH@g-C 3 N 4 @Ag 3 PO 4 Nanocomposite. ACS Omega.

[37] Luan N.H., Chang C.F. (2024). Fabrication of Ag3PO4/N-doped TiO2 nanotubes heterojunction photocatalysts for visible-light-driven photocatalysis. Chemosphere.

[38] Qian X., Xu Y., Xu Y. (2024). Bacterial cellulose based TiO2-CdS nanocomposite gel with enhanced photocatalytic activity for adsorptive degradation of cationic dye. Int. J. Biol. Macromol..

[39] Salmanzadeh-Jamadi Z., Habibi-Yangjeh A., Feizpoor S., Pourbasheer E., Chand H., Krishnan V., Wang C., Xie J., Zhong Y. (2022). Novel visible-light TiO2/Bi3O4Br photocatalysts with n-n heterojunction: Highly impressive performance for elimination of tetracycline and dye contaminants. Opt. Mater..

[40] Hilal Al Dhamri M., Maridevaru M.C., Sillanpaa M., Kim Y., Selvaraj R. (2024). Fabrication of chrysanthemums like TiO2@In2S3 S-scheme heterojunction nanostructures for the degradation of methylene blue dye present in aqueous solution. Inorg. Chem. Commun..

[41] Wang Q., Zhang W., Hu X., Xu L., Chen G., Li X. (2021). Hollow spherical WO3/TiO2 heterojunction for enhancing photocatalytic performance in visible-light. J. Water Process Eng..

[42] Shalini S., Sasikala T., Tharani D., Venkatesh R., Muthulingam S. (2024). Novel green CQDs/ZnO binary photocatalyst synthesis for efficient visible light irradiation of organic dye degradation for environmental remediation. J. Mol. Liq..

[43] Habibi M., Habibi-Yangjeh A., Pouran S.R., Chand H., Krishnan V., Xu X., Wang C. (2022). Visible-light-triggered persulfate activation by CuCo2S4 modified ZnO photocatalyst for degradation of tetracycline hydrochloride. Colloids Surf. A Physicochem. Eng. Asp..

[44] Shanmugam P., Ngullie R.C., Meejoo Smith S., Boonyuen S., Boddula R., Pothu R. (2023). Visible-light induced photocatalytic removal of methylene blue dye by copper oxide decorated zinc oxide nanorods. Mater. Sci. Energy Technol..

[45] Shoaib M., Naz M.Y., Shukrullah S., Munir M.A., Ghaffar A., Irfan M., Mursal S.N.F., Kamran K. (2022). Synthesis and testing of photocatalytic potential of MgFe2O4/ZnO/perlite magnetic heterojunction for degradation of synthetic dyes. Appl. Phys. A.

[46] Zabihi R., Monadi N. (2025). Fabrication of V doped zinc oxide-reduced graphene oxide magnetic heterojunction with enhanced sunlight driven photocatalytic performance. Mater. Today Commun..

[47] Shoaib M., Munir M.A., Naz M.Y., Abbas G., Irfan M., Rahman S., Ghanim A.A.J. (2023). Testing of Magnetic ZnO/MgFe2O4 Heterostructures for Photocatalytic Removal of Synthetic Dye Pollutants from Wastewater. Water Air Soil Pollut..

[48] Raza N., Raza W., Gul H., Kim K.H. (2021). ZnO–ZnTe hierarchical superstructures as solar-light-activated photocatalysts for azo dye removal. Environ. Res..

[49] Belousov A.S., Parkhacheva A.A., Shotina V.A., Titaev D.N., Suleimanov E.V., Shafiq I. (2024). Engineering a staggered type-II Bi2WO6/WO3 heterojunction with improved photocatalytic activity in wastewater treatment. Chemosphere.

[50] Tanveer M., Ali A.R., Qadeer M.A., Alam M., Cheema H.H., Hussain M.K., Farooq M.u.H., Shakil M., Naseem F., Nabi G. (2024). A novel composite (Bi2WO6/TiS2) presenting an excellent Z-scheme photocatalytic degradation for methylene blue dye under the visible light irradiation. Opt. Mater..

[51] Lai M.T.L., Lee K.M., Yang T.C.K., Lai C.W., Chen C.Y., Johan M.R., Juan J.C. (2023). Highly effective interlayer expanded MoS2 coupled with Bi2WO6 as p-n heterojunction photocatalyst for photodegradation of organic dye under LED white light. J. Alloys Compd..

[52] Chopade A.S., Kolhe N.D., Walekar L.S., Kadam A.N., Patil V.A., Al-Enizi A.M., Tamboli M.S., Mhamane D.S., Mali M.G. (2024). Chemical bath assisted construction of CuO/BiVO4 p-n heterojunction photocatalyst for visible light driven rapid removal of MB and Cr(VI). Colloids Surf. A Physicochem. Eng. Asp..

[53] Tran T.H., Le P.N.M., Ngo T.H., Huynh N.D.T., Oh W.C., Le M.V. (2024). An investigation on the visible light-driven Z-scheme BiVO4/g-C3N4 heterostructures: Performance, evaluation, and mechanism for dye and antibiotics degradation. Mater. Today Commun..

[54] Balasurya S., Das A., Alyousef A.A., Alqasim A., Almutairi N., Sudheer Khan S. (2021). Facile synthesis of Bi2MoO6-Ag2MoO4 nanocomposite for the enhanced visible light photocatalytic removal of methylene blue and its antimicrobial application. J. Mol. Liq..

[55] Xu D., Sun F., Liu F., Shao H., He W., Wang L., Ma Q., Yu W., Yu H., Dong X. (2024). 2D Bi2MoO6 nanosheets immobilized on electrospun 1D LaFeO3 hollow nanofibers: An efficacious direct Z-scheme p-n type heterojunction photocatalyst for removal of organic contaminants under visible-light irradiation. J. Environ. Chem. Eng..

[56] Nagar A., Basu S. (2022). Deciphering the role of β-phase Bi2O3 nanorods incorporated on g-C3N4 nanosheets for efficient pollutant removal. J. Phys. Chem. Solids.

[57] Goudarzi A., Rahemi N., Allahyari S. (2024). Plasma treatment of nano BiOBr-BiOI heterojunction for efficient photocatalytic performance under visible light. J. Photochem. Photobiol. Chem..

[58] Shoja A., Habibi-Yangjeh A., Mousavi M., Ghosh S. (2020). BiOBr and BiOCl decorated on TiO2 QDs: Impressively increased photocatalytic performance for the degradation of pollutants under visible light. Adv. Powder Technol..

[59] Zarezadeh S., Habibi-Yangjeh A., Mousavi M., Ghosh S. (2020). Synthesis of novel p-n-p BiOBr/ZnO/BiOI heterostructures and their efficient photocatalytic performances in removals of dye pollutants under visible light. J. Photochem. Photobiol. Chem..

[60] Zhang J., Sun X., Ma J., Yi Z., Xian T., Wang S., Liu G., Wang X., Yang H. (2023). Development of highly-efficient 0D/1D/0D dual Z-scheme CdS/ZnWO4/ZnS heterojunction photocatalysts in pollutant removal and involved mechanism. Appl. Surf. Sci..

[61] Wang Z., Li J., Fu S., Guo D., Tang J., Yang X., Xu R., Sui G., Chen S. (2023). Construction of MoS2/CdS/Bi2MoO6 Z-scheme photocatalyst for efficient photocatalytic degradation under visible-light. J. Solid State Chem..

[62] Madima N., Kefeni K.K., Kuvarega A.T., Mishra S.B., Mishra A.K. (2023). Visible-light-driven Z-scheme ternary Fe3O4/TiO2/g-C3N4 nanocomposite as reusable photocatalyst for efficient removal of dyes and chromium in water. Mater. Chem. Phys..

[63] Zhang X., Song Z., Yu X., Dong X., Peng Y., Wei K., Cao L., He X., Zhang Z., Fan J. (2023). Construction of heterogeneous structures of MIL-101(Fe)/Ce/g-C3N4 nanocomposites for enhanced photocatalytic activity under visible light. J. Solid State Chem..

[64] Huang H., Feng W., Niu Z., Qin X., Liu X., Shan B., Liu Y. (2022). Structural, optical and photocatalytic properties of magnetic recoverable Mn0.6Zn0.4Fe2O4@Zn0.9Mn0.1O heterojunction prepared from waste Mn–Zn batteries. J. Environ. Manage..

[65] Tahir N., Zahid M., Bhatti I.A., Jamil Y. (2022). Fabrication of visible light active Mn-doped Bi2WO6-GO/MoS2 heterostructure for enhanced photocatalytic degradation of methylene blue. Environ. Sci. Pollut. Res. Int..

[66] Cheng T., Zhu J., Chen C., Hu Y., Wu L., Zhang M., Cui L., Dai Y., Zhang X., Tian Y., Wu F. (2025). Construction of Advanced S-Scheme Heterojunction Interface Composites of Bimetallic Phosphate MnMgPO4 with C3N4 Surface with Remarkable Performance in Photocatalytic Hydrogen Production and Pollutant Degradation. Coatings.

[67] Yousef A., Zouli N.I., Maafa I.M., Hadidi H.M., Sallam S., Moosa M., El-Halwany M.M. (2022). Fabrication of heterojunction MnTiO3-TiO2-decorated carbon nanofibers via electrospinning as an effective multifunctional photocatalyst. Mater. Sci. Pol..

[68] Rana A., Sonu S., Soni V., Chawla A., Sudhaik A., Raizada P., Ahamad T., Thakur P., Thakur S., Singh P. (2025). Novel S-scheme derived Mo–Bi2WO6/WO3/Biochar composite for photocatalytic removal of Methylene Blue dye. J. Phys. Chem. Solids.

[69] Laxmiputra D.B.N., Girish Y., Nagarajaiah H., Udayabhanu, B Nityashree D., Pramoda K., Anush S.M., Pramoda K., Prashantha K., Nagarajaiah H. (2024). Construction of Z-Scheme MoS2/ZnFe2O4 heterojunction photocatalyst with enhanced photocatalytic activity under visible light. Mater. Res. Bull..

[70] Lahootifar Z., Habibi-Yangjeh A., Salmanzadeh-Jamadi Z., Khataee A. (2024). G-C3N4 tubes decorated with MnMoO4·H2O: Outstanding S-scheme photocatalyst for detoxification of water pollutants upon visible light. FlatChem.

[71] Liao D., Xu J., Liu C. (2023). Constructing MXene-derived Z-Scheme g-C3N4/Ti3C2TX/Ag3PO4 photocatalysts with enhanced charge transfer for aquatic organic pollutants removal. Colloids Surf. A Physicochem. Eng. Asp..

[72] Malik M., Ibrahim S.M., Nazir M.A., Tahir A.A., Tufail M.K., Shah S.S.A., Anum A., Wattoo M.A., Rehman A.u. (2023). Engineering of a Hybrid g-C3N4/ZnO-W/Cox Heterojunction Photocatalyst for the Removal of Methylene Blue Dye. Catalysts.

[73] Jiménez-Rangel K., Samaniego-Benítez J.E., Jiménez-Flores Y., Lartundo-Rojas L., Calderón H.A., Garduño-Wilches I., García-García A., Mantilla A. (2024). g-C3N4(p)/AgCl as photocatalyst for the simultaneous removal of Cr (VI) and methylene blue under visible light irradiation. J. Photochem. Photobiol. Chem..

[74] Kublik N., Gomes L.E., Plaça L.F., Lima T.H.N., Abelha T.F., Ferencz J.A.P., Caires A.R.L., Wender H. (2021). Metal-Free g-C3N4/Nanodiamond Heterostructures for Enhanced Photocatalytic Pollutant Removal and Bacteria Photoinactivation. Photochem.

[75] Shakir I. (2024). Synthesis of Gd doped BiFeO3/g-C3N4 composite: Enhancement of solar mediated photocatalytic performance. Mater. Sci. Eng., B.

[76] Su H.M., Vasu D., Chan S.Y., Liu Y.C., Jiang J., You Y.F., Chiu T.W., Chen S.C. (2024). Two-dimensional heterojunction layered graphene oxide/graphitic carbon nitride photocatalyst for removal of toxic environmental dye methylene blue. Environ. Pollut..

[77] Shanthini K., Anitha C., Alphonse N.R., Velmurugan K., Selvam V. (2023). GO-CNT/AgI nanocomposites: A facile synthesis and environmentally friendly method to removal of organic pollutants. J. Mol. Struct..

[78] Noman A.M.A., Alghobar M.A., Suresha S. (2021). Synthesis and activity evaluation of p-n CuO/CeO2-ZrO2 heterojunction photocatalyst for the removal of dye from industrial wastewater under visible light irradiation. J. Water Environ. Nanotechnol..

[79] Chopade A.S., Ransing A.A., Parale V.G., Mohite S.V., Kadam A.N., Kim J., Patil V.A., Gilani S.J., Tamboli M.S., Kim H.K. (2025). Improved charge carrier dynamics in rationally designed F-g-C3N4/In2O3 type II heterojunction for efficient pollutant removal: Mechanistic insights, real-world applications, and toxicity study. J. Water Process Eng..

[80] Xu G., Du M., Zhang J., Li T., Guan Y., Guo C. (2022). Facile fabrication of magnetically recyclable Fe3O4/BiVO4/CuS heterojunction photocatalyst for boosting simultaneous Cr(VI) reduction and methylene blue degradation under visible light. J. Alloys Compd..

[81] Shi K., Wang Z., Luo J., Tian Z., Zhu Y., Yao H. (2025). Interfacial chemically bonding of 0D/2D FeOOH QDs/CuFe2O4 S-type heterojunction to boost photocatalytic degradation of methylene blue and tetracycline. J. Environ. Chem. Eng..

[82] Guo R.F., Liang P., Li X.Y., Liu Z.H. (2021). Fabrication of a dual Z-scheme GACN/NiO/Ni3(BO3)2 composite with excellent photocatalytic activity for methylene blue and tetracycline removal. Sep. Purif. Technol..

[83] Zhong X., Li Y., Liu S., Liu Q., Yang K., Li X., Jin W., Liu H., Xie R. (2025). Nitrogen vacancy mediated g-C3N4/BiVO4 Z-scheme heterostructure nanostructures for exceptional photocatalytic performance. Environ. Res..

[84] Zhao J., Deng Q., Yang Z., Zhang H., Zhang C. (2024). Synthesis of novel g-C3N4/CaTiO3/CQDs heterojunction photocatalyst with enhanced visible light photocatalytic performance. Opt. Mater..

[85] Leelavathi H., Muralidharan R., Abirami N., Tamizharasan S., Sankeetha S., Kumarasamy A., Arulmozhi R. (2023). Construction of step-scheme g-C3N4/Co/ZnO heterojunction photocatalyst for aerobic photocatalytic degradation of synthetic wastewater. Colloids Surf. A Physicochem. Eng. Asp..

[86] Sun J., Lin X., Xie J., Zhang Y., Wang Q., Ying Z. (2020). Facile synthesis of novel ternary g-C3N4/ferrite/biochar hybrid photocatalyst for efficient degradation of methylene blue under visible-light irradiation. Colloids Surf. A Physicochem. Eng. Asp..

[87] Zhang W., Ma Y., Zhu X., Liu S., An T., Bao J., Hu X., Tian H. (2021). Fabrication of Ag decorated g-C3N4/LaFeO3 Z-scheme heterojunction as highly efficient visible-light photocatalyst for degradation of methylene blue and tetracycline hydrochloride. J. Alloys Compd..

[88] Shenoy S., Tarafder K., Sridharan K. (2020). Graphitic C3N4/CdS composite photocatalyst: Synthesis, characterization and photodegradation of methylene blue under visible light. Phys. B Condens. Matter.

[89] Zhao J., Cao X., Bai Y., Chen J., Zhang C. (2023). Simple synthesis of CaTiO3/g-C3N4 heterojunction for efficient photodegradation of methylene blue and levofloxacin. Opt. Mater..

[90] Cheng T., Gao H., Liu G., Pu Z., Wang S., Yi Z., Wu X., Yang H. (2022). Preparation of core-shell heterojunction photocatalysts by coating CdS nanoparticles onto Bi4Ti3O12 hierarchical microspheres and their photocatalytic removal of organic pollutants and Cr(VI) ions. Colloids Surf. A Physicochem. Eng. Asp..

[91] Ren J.X., Zhu J.L., Shi S.C., Yin M.Q., Huang H.D., Li Z.M. (2022). In-situ structuring a robust cellulose hydrogel with ZnO/SiO2 heterojunctions for efficient photocatalytic degradation. Carbohydr. Polym..

[92] Ning D., Li J., Lan Y., Sohn H.Y., Yang J., Chen C., Chu Z., Mao X. (2023). Molten salt synthesis of Z-scheme CeO2/C3N4 photocatalysts with excellent properties for removal of organic pollutants: Characterization, kinetics and mechanisms. J. Rare Earths.

[93] Zhang M., Zhu R., Deng B., Huang Y., Ren H. (2025). Z-Scheme CeO2@PDA/BiOBr heterojunction with PDA electronic transfer medium for photocatalytic elimination of methylene blue and tetracycline. J. Mol. Struct..

[94] Rahman T.U., Roy H., Shoronika A.Z., Fariha A., Hasan M., Islam M.S., Marwani H.M., Islam A., Hasan M.M., Alsukaibi A.K.D. (2023). Sustainable toxic dye removal and degradation from wastewater using novel chitosan-modified TiO2 and ZnO nanocomposites. J. Mol. Liq..

[95] Abubshait S.A., Iqbal S., Abubshait H.A., AlObaid A.A., Al-Muhimeed T.I., Abd-Rabboh H.S.M., Bahadur A., Li W. (2021). Effective heterointerface combination of 1D/2D Co-NiS/S-g-C3N4 heterojunction for boosting spatial charge separation with enhanced photocatalytic degradation of organic pollutants and disinfection of pathogens. Colloids Surf. A Physicochem. Eng. Asp..

[96] Reza Amani-Ghadim A., Dadkhah S., Abdouss M., Khataee A., Sattari S., Fattahi M. (2024). Development of a novel Z-scheme CoxNi1−xTiO3/CdS (x = 0.5) photocatalyst for the efficient degradation of organic pollutants via a visible-light-driven photocatalytic process. J. Colloid Interface Sci..

[97] Derikvand H., Tahmasebi N., Barzegar S. (2024). Construction of a direct Z-scheme Cs3Bi2Cl9/g-C3N4 heterojunction composite for efficient photocatalytic degradation of various pollutants in water: Performance, kinetics and degradation mechanism. Chemosphere.

[98] Shiekhmohammadi A., Alamgholiloo H., Asgari E., Jalilzadeh Z. (2025). A plasmonic S-scheme Ag/ZrO2/TCN photocatalyst for enhancing interfacial charge transfer: Insights to machine learning models and mechanism for photodegradation. Colloids Surf. A Physicochem. Eng. Asp..

[99] Liu W., Zhou M., Fu H. (2023). Design of Ag@ZnO@Ti3C2 MXene heterojunction photocatalyst for enhanced photocatalytic degradation activity of methylene blue and levofloxacin under visible light irradiation. J. Environ. Chem. Eng..

[100] Adday A.S., Al-Jubouri S.M. (2024). Photocatalytic oxidative removal of the organic pollutant from wastewater using recyclable Ag2O@CRA heterojunction photocatalyst. Case Stud. Chem. Environ. Eng..

[101] Elgorban A.M., Al Kheraif A.A., Syed A. (2021). Construction of Ag2WO4 decorated CoWO4 nano-heterojunction with recombination delay for enhanced visible light photocatalytic performance and its antibacterial applications. Colloids Surf. A Physicochem. Eng. Asp..

[102] Janani B., Syed A., Raju L.L., Bahkali A.H., Al-Rashed S., Elgorban A.M., Ahmed B., Thomas A.M., Khan S.S. (2022). Designing intimate porous Al2O3 decorated 2D CdO nano-heterojunction as enhanced white light driven photocatalyst and antibacterial agent. J. Alloys Compd..

[103] Wang Q., Zhu S., Zhao S., Li C., Wang R., Cao D., Liu G. (2022). Construction of Bi-assisted modified CdS/TiO2 nanotube arrays with ternary S-scheme heterojunction for photocatalytic wastewater treatment and hydrogen production. Fuel.

[104] Luo C., Liao M., Xiong J., Wang L., Zeng H. (2025). Fabrication of Bi/S-Bi2O2CO3 plasmon heterojunction with sulfur-activated enhancement for photocatalysis. Colloids Surf. A Physicochem. Eng. Asp..

[105] Liu F., Wang Y., Xu D., Sun F., Zhang S., Wang W., Li X., Yu W., Yu H., Dong X. (2023). Full-spectrum-responsive 1D/2D BiVO4:Er3+,Yb3+/BiOCl core-shell S-scheme heterostructure with boosted charge transport and redox capacity for the efficient removal of organic pollutants. Ceram. Int..

[106] Akechatree N., Rajendran R., Rojviroon T. (2025). Development of Z-scheme BiVO 4/g-C 3 N 4/rGO heterojunction nanocomposite for enhanced photocatalytic degradation and antibacterial activity. Mater. Res. Bull..

[107] Xu Q., Zhang L., Yu J., Wageh S., Al-Ghamdi A.A., Jaroniec M. (2018). Direct Z-scheme photocatalysts: Principles, synthesis, and applications. Mater. Today.

[108] Wenderich K., Mul G. (2016). Methods, Mechanism, and Applications of Photodeposition in Photocatalysis: A Review. Chem. Rev..

[109] Low J., Jiang C., Cheng B., Wageh S., Al-Ghamdi A.A., Yu J. (2017). A Review of Direct Z-Scheme Photocatalysts. Small Methods.

[110] Che L., Pan J., Cai K., Cong Y., Lv S.-W. (2023). The construction of p-n heterojunction for enhancing photocatalytic performance in environmental application: A review. Sep. Purif. Technol..

[111] Balapure A., Ray Dutta J., Ganesan R. (2024). Recent advances in semiconductor heterojunctions: a detailed review of the fundamentals of photocatalysis, charge transfer mechanism and materials. RSC Appl. Interfaces.

[112] Kumari P., Bahadur N., Kong L., O’Dell L.A., Merenda A., Dumée L.F. (2022). Engineering Schottky-like and heterojunction materials for enhanced photocatalysis performance – a review. Mater. Adv..

[113] Yang Y., Zeng Z., Zeng G., Huang D., Xiao R., Zhang C., Zhou C., Xiong W., Wang W., Cheng M. (2019). Ti3C2 Mxene/porous g-C3N4 interfacial Schottky junction for boosting spatial charge separation in photocatalytic H2O2 production. Appl. Catal. B Environ..

[114] Chen X., Pan W.g., Guo R.t., Hu X., Bi Z.x., Wang J. (2022). Recent progress on van der Waals heterojunctions applied in photocatalysis. J. Mater. Chem. A..

[115] Li J., Zhang Z., Cui W., Wang H., Cen W., Johnson G., Jiang G., Zhang S., Dong F. (2018). The Spatially Oriented Charge Flow and Photocatalysis Mechanism on Internal van der Waals Heterostructures Enhanced g-C 3 N 4. ACS Catal..

[116] Jia W., Ding B., Qian X., Yang Y., Mao L., Cai X., Guo S., Zhang J. (2021). van der Waals g-C 3 N 4/BiLuWO 6 Heterojunctions from Theoretical Predictions to Photocatalytic Applications. J. Phys. Chem. C.

[117] Kaushal S., Kurichh P., Singh P.P. (2021). Novel 3D flower like ZnO/MnV2O6 heterojunction as an efficient adsorbent for the removal of imidacloprid and photocatalyst for degradation of organic dyes in waste water. Polyhedron.

[118] Ojeda L., Deaquino-Garcia V., Castillo P.C.H.-D., Padmasree K.P., Esquivel-Castro T.A., Garcés-Patiño L.A., Flores-Saldaña D.S.M., Ojeda-Galvan H.J., Oliva J. (2025). Increasing the surface defects/redox species in LaFe0.94V0.01Mn0.05O3/graphene electrodes to enhance the energy density of supercapacitors and the photocatalytic degradation of methylene blue dye. Diam. Relat. Mater..

[119] Valadez-Renteria E., Garcia C.R., Hernandez del Castillo P.C., Paniagua-Chavez M.L., Garcés-Patiño L.A., Oliva J. (2026). Effect of surface defects in graphitic compounds recovered from spent alkaline/Lithium-ion batteries on the solar photodegradation of methylene blue dye. Surf. Interfaces.

[120] Rosales M., Oliva J., Rodríguez-Gonzalez C., Silva-Vidaurri L.G., Salas P. (2026). Dual-Function MnBi2O4: Ni for Flexible Supercapacitors and Sunlight-Driven Degradation of Organic Pollutants. Korean J. Chem. Eng..

[121] Erdem B., Sevinç S., Erdem S., Öksüzoğlu R.M. (2023). Comparative influence of adsorption assisted magnetic mesoporous TiO2 photocatalyst for the removal of methylene blue and rhodamine B. React. Kinet. Mech. Catal..

[122] Juliya A.P., Abdul Mujeeb V.M., Jayaram P. (2023). Sunlight assisted photocatalytic degradation of methylene blue via mesoporous TiO2/RuO2 binary nanosystem. Mater. Lett..

[123] Sadikin S.N., Ridwan J., Umar M.I.A., Raub A.A.M., Yunas J., Hamzah A.A., Dahlan D., Rahman M.Y.A., Umar A.A. (2023). Photocatalytic activity and stability properties of porous TiO2 film as photocatalyst for methylene blue and methylene orange degradation. Int. J. Electrochem. Sci..

[124] Zulfa L.L., Hidayat A.R.P., Utomo W.P., Putri D.R., Hartanto D., Widyastuti W., Ali B.T.I., Akhlus S., Ediati R. (2025). Fabrication of mixed-ligand ZIF-8-derived mesoporous ZnO for enhanced photocatalytic activities for methylene blue and naphthol degradation. J. Mol. Struct..

[125] Albadri A.E.A.E., Aissa M.A.B., Modwi A., Saleh S.M. (2023). Synthesis of Mesoporous Ru-ZnO@g-C3N4 Nanoparticles and Their Photocatalytic Activity for Methylene Blue Degradation. Water.

[126] Panda P.K., Pattanaik R., Mishra S., Pradhan D., Kumar Dash S. (2023). Superior photocatalytic degradation of MB dye using BiVO4 nanoparticles under solar light irradiation. Mater. Today Proc..

[127] Cai Y., Yang X., Li Y., Ling R., Sun G. (2024). Preparation and effects of calcining temperature and pH on the photocatalytic activity of BiVO4 microcrystal for degrading methylene blue. Ionics.

[128] Sirusy S., Ashrafi H., Akhond M. (2023). Microtubular Nanoporous g-C 3 N 4 @Ag as an Efficient Photocarrier Transfer Agent and Visible Light-Harvesting for Photodegradation of Methylene Blue. ACS Appl. Nano Mater..

[129] Salve V., Agale P., Balgude S., Mardikar S., Dhotre S., More P. (2025). Enhanced photocatalytic activity of SnO 2 @g-C 3 N 4 heterojunctions for methylene blue and bisphenol-A degradation: effect of interface structure and porous nature. RSC Adv..

[130] Liu X., Ma S., Yang H., He P., Duan X., Jia D., Colombo P., Zhou Y. (2024). 3D-printed green and low-cost porous g-C 3 N 4/geopolymer components for the removal of methylene blue from wastewater. J. Am. Ceram. Soc..

[131] Najibikhah P., Shayesteh H., Rahbar-Kelishami A. (2025). Two-dimensional Ti3C2Tx MXene/MOF composite for enhanced methylene blue adsorption. J. Mol. Liq..

[132] Zhang W., Li Z., Ding Y., Zhang P., Wan Y., Zhang Q., Ma J., Wang Y. (2026). Bimetallic MOFs/MXene-derived heterogeneous photocatalyst for enhanced adsorption and synergistic photo-Fenton degradation of methylene blue. Sustain. Mater. Technol..

[133] González S., Jaramillo-Fierro X. (2025). Density Functional Theory Study of Methylene Blue Demethylation as a Key Step in Degradation Mediated by Reactive Oxygen Species. Int. J. Mol. Sci..

[134] Houas A. (2001). Photocatalytic degradation pathway of methylene blue in water. Appl. Catal. B Environ..

[135] Rauf M.A., Meetani M.A., Khaleel A., Ahmed A. (2010). Photocatalytic degradation of Methylene Blue using a mixed catalyst and product analysis by LC/MS. Chem. Eng. J..

[136] Ahmadi B., Fallah A., Ghamarpoor R., Jamshidi M. (2025). Methylene blue beyond the dye: A critical review on its role as a benchmark pollutant in photocatalyst design, Results. Chem.

